# The race that segments a nation: Findings from a convenience poll of attitudes toward the Melbourne Cup Thoroughbred horse race, gambling and animal cruelty

**DOI:** 10.1371/journal.pone.0248945

**Published:** 2021-03-24

**Authors:** Bethany J. Wilson, Kirrilly R. Thompson, Paul D. McGreevy

**Affiliations:** 1 Sydney School of Veterinary Science, Faculty of Science, University of Sydney, Sydney, NSW, Australia; 2 University of South Australia Business School, Adelaide, SA, Australia; Massey University, NEW ZEALAND

## Abstract

The annual Melbourne Cup Thoroughbred horse race has iconic status among many Australians but sits in the context of increasing criticism of the welfare of Thoroughbred racing horses and the ethics of gambling. Despite heated debates and protests playing out in the public domain, there is scant empirical research to document Australian attitudes to the Melbourne Cup, or horse racing more generally. Specifically, little is known about how support for or against the Melbourne Cup correlate with age, gender, income and level of education. To provide a more nuanced understanding of attitudes towards the cup beyond the rudimentary binaries of those who are ‘for’ or ‘against’ gambling and horse racing, the purpose of the study was to identify clusters of people with particular views. An opportunistic survey collected data on respondents’ gender, age, place of residence, weekly income, employment status and highest level of education, and sought their level of agreement with six statements about the Melbourne Cup, gambling and animal cruelty. Ordinal logistic regression and Chi-square analysis were used to evaluate the age and gender of respondents in clusters respectively. Agreement with the statements revealed some significant associations. Male respondents were at greater odds for agreement with the statement: *I regularly bet on horse races* (OR = 2.39; 95% CI = 1.78–3.22) as were respondents aged 18–19 years (OR = 2.88; 95% CI = 1.13–7.35) and 20–24 years (OR = 1.90; 95% CI 1.00–3.62) compared with the median 35–40 years age bracket. Agreement with the statement: I will watch the Melbourne Cup but will not place a bet was more likely among the full-time employed (OR = 1.60; 95% CI = 1.10–2.32), for those aged 20–24 years (OR = 1.85; 95% CI = 1.16–2.95). The odds of increasing agreement with the statement: *I have never been interested in the Melbourne Cup* were multiplied by 0.87 (95% CI = 0.82–0.92) with each successive five-year age bracket. The most useful of the predictor variables for agreement was level of education. The odds of increasing with the statement: *I have become less interested in the Melbourne Cup over recent years because of my concerns with gambling* were multiplied by 1.09 (95% CI = 1.02–1.15) for each increased level of education. Agreement with the statement: *I have become less interested in the Melbourne Cup because of my concerns about animal cruelty* was weaker amongst male respondents (OR = 0.62; 95% CI = 0.48–0.80), and those in increasing age brackets (OR = 0.88; 95% CI = 0.83–0.93). A series of six clusters were identified that show how certain attributes of respondents characterise their responses. The authors labelled these clusters “Devotees” (n = 313; 30.4% of respondents), “Flaneurs” (n = 244; 21.8% of respondents), “Disapprovers” (n = 163; 15.9% of respondents), “Casuals” (n = 148; 14.4% of respondents), “Gamblers” (n = 126; 12.3% of respondents) and “Paradoxical-voters” (n = 54; 5.3% of respondents). The implications for support of the Melbourne Cup are explored.

## 1. Introduction

The Melbourne Cup is a Thoroughbred horse race which takes place on the first Tuesday of November every year in the Melbourne suburb of Flemington, as the premier event of the Melbourne Spring Carnival [1]. First run in 1861, the race has become both a prominent part of the Australian national culture, listed with barbeques, football and ANZAC day as a core cultural symbol of Australian identity [2] and also a significant event on the global racing calendar, comparable to the Grand National, Kentucky Derby and Japan Cup [3, 4].

Growing from the estimated crowd of four thousand who attended the first Melbourne Cup day [2] to turn-outs well in excess of 100,000 in the modern era [1, 4], the event contributes an estimated AUD350 million to the state economy [4]. Annual betting of more than AUD105 million on this single race has been recorded, and global television audiences have been estimated at more than 1 billion [4]. In addition to gambling and the sport of horse-racing itself, since the 1960s the Melbourne Cup has also become intimately associated with fashion, and celebrity culture, creating another face of horse-racing with which the public can engage [2, 5].

But despite its economic and social benefits, Thoroughbred racing in general [6], and the Melbourne Cup day in particular, potentially carry significant welfare costs to both horses and people. In addition to high profile deaths, such as the euthanasia of racehorse Cliffsofmoher following an injury early in the 2018 Melbourne Cup and the sudden death of Admire Rakti shortly after racing in 2014, In addition to the high profile deaths of Melbourne Cup runners on track or shortly after (7 horses since 2013), Thoroughbred racing is associated with widespread wastage [5] and acute and chronic pain from musculoskeletal injuries [7], pulmonary haemorrhages [8], gastric ulcers [9] and increasing public distaste for the use and consequence of equipment such aswhips [10–12] and tongue-ties [13]. Additionally, problem gambling is a widespread financial and mental health issue among Australians [14]. These concerns can have significant implications for the Thoroughbred racing industry’s social license to operate [15, 16]. Horse racing has been increasingly controversial in Australia over the past decades, mostly in relation to whip use [17–20] and injury and fatality rates in jumps racing [21–23]. More recently, there was a shared outcry from racing proponents and opponents alike in response to a *7*.*30 Report* exposé into the end of life for horse ‘wastage’ from the Australian Thoroughbred racing industry, particularly horses which had raced in New South Wales [24].

The ethical use of horses demands that we consider the welfare impact that horse-racing has on horses despite the economic and social benefits of horse-racing [25–27]. The Melbourne Cup, despite (or perhaps because of) its status as a cultural icon [28], is no exception.

Ahead of the 2018 Melbourne Cup, a commercial poll of adult Australians revealed that, when asked about horse-racing in general, 8% professed high interest in the sport, 20% reported moderate interest, while 70% said they had low or no interest [29]. However, although only 19% of the sample reported they regularly bet on horse-races, 38% said they would be watching the Melbourne Cup that year and would place a bet. Furthermore, 33% said they would be watching the event but not placing a bet. These data seem to confirm the iconic status that the Melbourne Cup has for many Australians [29].

Following the example offered by an earlier report on the use of polling data from a third party [20], the current study returns to the original data to explore relationships among these attitudes and respondents’ income, employment status, age and sex. It also explores how attitudes toward the Melbourne Cup intersect with concerns about animal welfare concerns and problem gambling.

## 2. Materials and methods

### 2.1. Data

The data collection was performed by Essential Research, a division of Essential media, who, in addition to demographic data about gender_,_ age_,_ place of residence, weekly income, employment status and highest level of education, electronically polled respondents for their level of agreement with the following six statements:

1. I regularly bet on horse races
2. I rarely bet on horse races but will be watching the Melbourne Cup and placing a bet
3. I will watch the Melbourne Cup but will not place a bet
4. I have never been interested in the Melbourne Cup
5. I have become less interested in the Melbourne Cup over recent years because of my concerns with gambling
6. I have become less interested in the Melbourne Cup because of my concerns about animal cruelty

The questions were asked online as part of a larger omnibus of questions on a variety of topics allowing several days for survey completion under the supervisions of members of the Australian Market and Social Research Society (AMSRS), acting under a professional code of behavior. This was undertaken in the fortnight prior to 6th November 2018. We note that these data were collected well before the profile of Australian Racing was challenged by documentaries such as The Final Race (ABC TV’s 7.30 Report, 17^th^ of October 2019). While the process is intended to sample a random sample of the population, sampling errors due to lack of 100% response rate of invited respondents and gaps in coverage of the original pool from which invited respondents were sourced cannot be ruled out.

### 2.2. Analysis

#### 2.2.1. Demographics

The association between respondents and respondent demographics were explored by ordinal logistic regression using the polr function of the MASS package in R [30, 31].

The model used was

Score_ij_ ~ Gender_i_+ Age_i_+ Residence_i_+ Income_i_+ Employment_i_+ Education_i_

Where Score_ij_ = the Agreement Score (ie Strongly Disagree< Disagree<Don’t know<Agree< Strongly Agree) of Participant i to statement j (where j is one of statements 1–6 above); Gender _I_ = Whether Participant i identified as “Male” or “Female”; Age_i_ = the age in years bracket nominated by Participant i for themselves; Residence_i_ = Civic area where Participant i said that they lived; Income_i_ = Participant i’s nominated weekly income bracket; Employment_i_ = Participant i’s nominated employment status; Education_i_ = Participant I’s highest stated level of education.

#### 2.2.2. Cluster analysis

A hierarchical cluster analysis was performed on the Agreement Scores of the six statements (using Gower distance) with the daisy and hclust functions [32]. “Don’t know” was again placed centrally (i.e., Strongly Disagree< Disagree<Don’t know<Agree< Strongly Agree).

Ordinal logistic regression and Chi-square analysis were used to evaluate the age and gender of respondents in clusters respectively.

## 3. Results

### 3.1. Demographics

A total of 1028 respondents completed the survey, of whom 526 (51.2%) were female and 502 (48.8%) were male. Their agreement with the six statements about the Melbourne Cup, gambling and horse racing were stratified by gender (See Table 1).

**Table 1:** Ordinal agreement among 1028 respondents with six statements regarding the Melbourne Cup and horse-racing stratified by gender.

Statement	Strongly Disagree	Disagree	Don’t Know	Agree	Strongly Agree
S1: I regularly bet on horse races					
Female	348 (58%)	120 (56%)	16 (44%)	31 (28%)	11 (17%)
Male	255 (42%)	96 (44%)	20 (56%)	78 (72%)	53 (83%)
**Total**	**603 (59%)**	**216 (21%)**	**36 (4%)**	**109 (11%)**	**64 (6%)**
S2: I rarely bet on horse races but will be watching the Melbourne Cup and placing a bet					
Female	202 (54%)	98 (47%)	39 (54%)	119 (49%)	68 (51%)
Male	169 (46%)	111 (53%)	33 (46%)	123 (51%)	66 (49%)
**Total**	**371 (36%)**	**209 (20%)**	**72 (7%)**	**242 (24%)**	**134 (13%)**
S3: I will watch the Melbourne Cup but will not place a bet					
Female	189 (54%)	138 (52%)	47 (55%)	104 (45%)	48 (48%)
Male	158 (46%)	126 (48%)	39 (45%)	126 (55%)	53 (52%)
**Total**	**347 (34%)**	**264 (26%)**	**86 (8%)**	**230 (22%)**	**101 (10%)**
S4: I have never been interested in the Melbourne Cup					
Female	154	182	25	78	87
Male	139	159	27	108	69
**Total**	**293 (29%)**	**341 (33%)**	**52 (5%)**	**186 (18%)**	**156 (15%)**
S5: I have become less interested in the Melbourne Cup over recent years because of my concerns with gambling					
Female	187	179	40	67	53
Male	164	154	38	102	44
**Total**	**351 (34%)**	**333 (32%)**	**78 (8%)**	**169 (16%)**	**97 (9%)**
*S6*: *I have become less interested in the Melbourne Cup because of my concerns about animal cruelty*					
Female	148	164	41	85	88
Male	178	168	35	78	43
**Total**	**326 (32%)**	**332 (32%)**	**76 (7%)**	**163 (16%)**	**131 (13%)**

Frequency of response and (%) are offered. Total rows sum to 100% horizontally and each sub category, divided by gender, will sum vertically”.

**Statement 1: “I regularly bet on horse races”.** Male respondents were at greater odds than the average respondent for agreement with this statement (OR = 2.39; 95% CI = 1.78–3.22), as were respondents aged 18–19 years (OR = 2.88; 95% CI = 1.13–7.35) and 20–24 years (OR = 1.90; 95% CI 1.00–3.62) compared with the median 35–40 years age bracket, and income earners in the bracket AUD$1-$199 per week (OR = 3.07; 95% CI 1.26–7.47).

In contrast, female respondents, respondents earning in the range of AUD$1,250-$1,499 per week (OR = 0.54; 95% CI = 0.32–0.91) and students (OR = 0.35; 95% CI = 0.16–0.79) were at lesser odds than average for agreement. Respondents over 64 years of age (OR = 0.34 95% CI = 0.16–0.73) were in less agreement than the median 35–40 years age bracket. Testing for an interaction between Gender and Age was found it to be not significant (p = 0.43).

**Statement 2: “I rarely bet on horse races but will be watching the Melbourne Cup and placing a bet”.** Unlike the first statement about habitual gambling, male respondents were not significantly more likely to say that, although they rarely bet on horse-racing, they would bet on the Melbourne Cup (LR χ^2^ = 0.0382, df = 1 p = 0.85).

Those aged 20–24 years showed higher odds of agreement (OR = 1.73; 95% CI = 1.10–2.71) with this statement than average whereas those aged 30–34 (OR = 0.64; 95% CI = 0.44–0.94) showed lower agreement. Respondents in the AUD$1,250-$1,499 per week income range were at lower odds of intending to gamble on the Melbourne Cup, as they also were at lower odds of regular gambling (OR = 0.58; 95% CI = 0.38–0.89). Those employed full- (OR = 1.98; 95% CI = 1.36–2.89) and part-time (OR = 1.49; 95% CI = 1.01–2.20) were at increased odds compared to the average of agreement for this statement.

**Statement 3: “I will watch the Melbourne Cup but will not place a bet”.** Agreement with this statement was more likely among the full-time employed (OR = 1.60; 95% CI = 1.10–2.32), for those aged 20–24 years (OR = 1.85; 95% CI = 1.16–2.95) and less likely for those aged 50–54 years (OR = 0.67; 95% CI = 0.47–0.96).

**Statement 4: “I have never been interested in the Melbourne Cup”.** The odds of agreement with this statement were highest among the relatively young age brackets 25–29 years (OR = 2.04; 95% CI = 1.40–2.97) and 30–34 (OR = 1.61; 95% CI = 1.10–2.35) and lowest in the older 60–64 years bracket (OR = 0.59; 95% CI = 0.38–0.92) compared to the average and in the 65 or older range 34 (OR = 0.40; 95% CI = 0.20–0.77) when compared to the 35–39 years range. This pattern is also true if age brackets are modelled ordinally with the odds of increasing agreement with the statement being multiplied by 0.87 (95% CI = 0.82–0.92) with each successive five-year age bracket. Household income in the bracket AUD$600-$799 per week also significantly reduced the odds of agreement compared to average (OR = 0.59; 95% CI = 0.38–0.92).

**Statement 5: “I have become less interested in the Melbourne Cup over recent years because of my concerns with gambling”.** The most useful of the predictor variables for this statement was level of education. When education was modelled ordinally, the odds of increasing agreement with this statement were multiplied by 1.09 (95% CI = 1.02–1.15) for each increased level of education.

The odds of agreement were lowered for those in the income bracket of AUD$800-$999 per week. (OR = 0.55; 95% CI = 0.36–0.83).

**Statement 6: “I have become less interested in the Melbourne Cup because of my concerns about animal cruelty”.** Male respondents (OR = 0.62; 95% CI = 0.48–0.80), and increasing age brackets (OR = 0.88; 95% CI = 0.83–0.93) were associated with lower odds of agreement with this statement as did the AUD$800-$999 per week household income bracket (OR = 0.57; 95% CI = 0.38–0.87).

### 3.2. Cluster analysis

**Fig 1:**
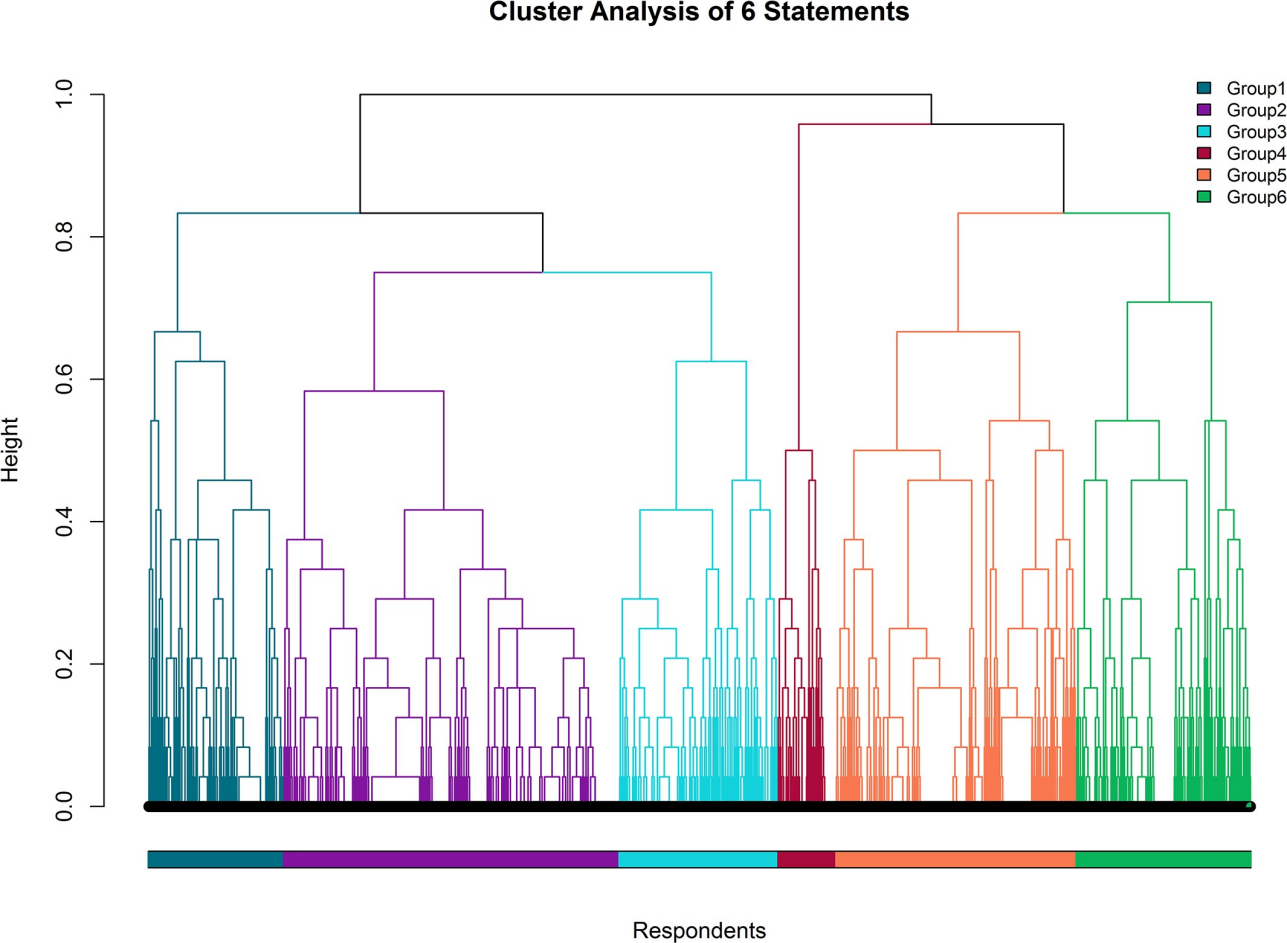
Cluster analysis of respondents (n = 1028) by ordinal agreement with six statements regarding 1028 respondents’ attitudes to the Melbourne Cup.

Respondents were classified into six groups through agglomerative hierarchical clustering based on the Gower Distance. The hierarchical relationship between these six groups is shown by the dendrogram in Fig 1.

**Fig 2:**
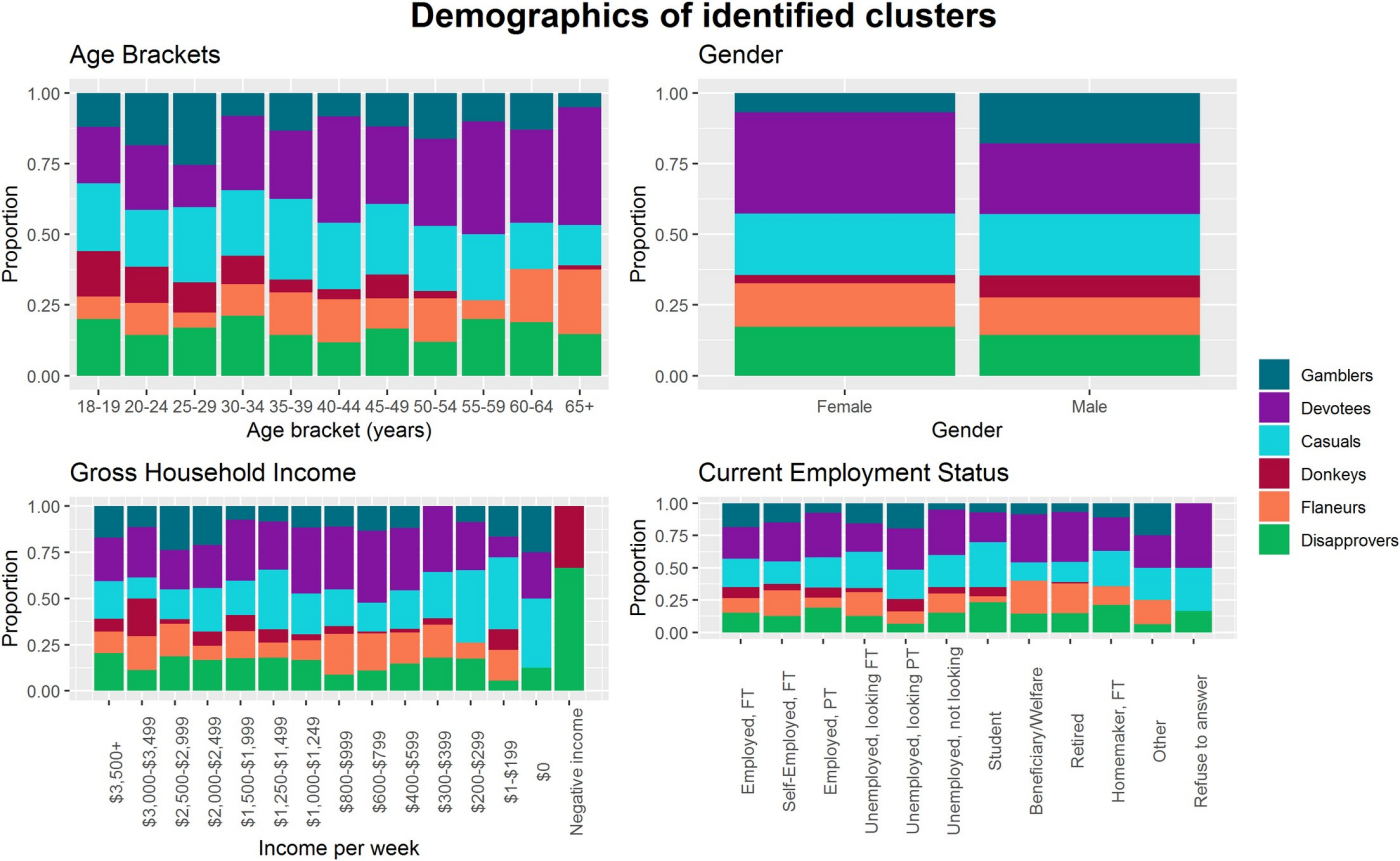
Demographics of respondents (n = 1028) assigned to six clusters by ordinal agreement with six statements regarding attitudes to the Melbourne Cup.

The demographics of the clusters are shown in Fig 2.

#### 3.2.1. Clusters

The six clusters are described below in order from most to least represented within the sample.

**“Devotees”.** This cluster included 313 (30.4%) respondents. These respondents did not report regular gambling on horse-races (99.7% disagree or strongly disagree with “I regularly bet on horses”). Nevertheless, they showed very high interest in the Melbourne Cup (99.4% disagreed or strongly disagreed with “I have never been interested in the Melbourne Cup”) and many planned to bet on it (63.6% agreed or strongly agreed with “I rarely bet on horse races but will be watching the Melbourne Cup and placing a bet”). Very few of this group reported reduced interest in the Cup due to gambling or welfare concerns (99.4% disagreed or strongly disagreed with “I have become less interested in the Melbourne Cup over recent years because of my concerns with gambling “; 97.8% disagreed or strongly disagreed with “I have become less interested in the Melbourne Cup over recent years because of my concerns about animal cruelty “). Women were over-represented among Devotees (χ^2^ = 7.2755, df = 1, p-value = 0.007).

**“Flaneurs”.** This cluster included 224 (21.8%) respondents. Flaneurs did not report high rates of regular gambling on horse races (82.6% disagreed or strongly disagreed with “I regularly bet on horses”) and they reported relatively low intention of watching the Melbourne Cup and placing a bet (16.1% agreed or strongly agreed with “I rarely bet on horse races but will be watching the Melbourne Cup and placing a bet”. They showed relatively low interest in the Melbourne Cup (79% agreed or strongly agreed with “I have never been interested in the Melbourne Cup”, and only 3.6% disagreed or strongly disagreed). Few agreed or strongly agreed to having reduced interest in the Melbourne Cup due to concerns about gambling (6.7%), but more reported reduced interest due to animal welfare concerns (17.9%). Neither women nor men were significantly over-represented but respondents in this cluster were younger than Devotees (-0.70, SE = 0.15, p<0.01).

**“Disapprovers’”.** This cluster included 163 (15.9%) respondents. Disapprovers did not report regular gambling on horse races (98.2% disagreed or strongly disagreed with “I regularly bet on horses”). Less than a quarter of this group agreed that they were planning to watch the Cup, with (22.1%) or without betting (16.0%). Neither women nor men were significantly over-represented but respondents in this cluster were younger than Devotees (-0.49, SE = 0.17, p<0.01). Some Disapprovers revealed apostatic views. They reported the greatest loss of interest in the Melbourne Cup due to moral and ethical concerns; 89.0% reported lessened interest due to concerns with gambling, and 74.2% due to concerns with animal cruelty. A reasonable number of respondents in this cluster revealed dissenting views, as 35.6% disagreed or strongly disagreed that they have never been interested in the Melbourne Cup.

**“Casuals”.** This cluster included 148 (14.4%) respondents. Like the Devotees, these respondents did not report regular gamblers on horse races (100% disagree or strongly disagree with “I regularly bet on horses”). Nonetheless, they did show high interest in the Melbourne Cup (89.2% disagreed or strongly disagreed with “I have never been interested in the Melbourne Cup”), but they do not generally plan to bet on it (86.5% agreed or strongly agreed with “I will watch the Melbourne Cup but will not place a bet”). About a third of these respondents reported reduced interest in the Cup due to concerns about animal welfare (33.8%) and slightly fewer due to concerns about gambling (31.1%). This cluster was not significantly older or younger than the Devotees and neither women nor men were overrepresented.

**“Gamblers”.** This cluster included 126 (12.3%) respondents. Gamblers tended to report high levels of betting on horses in general (88.1% agree or strongly agree with “I regularly bet on horses”). They showed high interest in the Melbourne Cup (94.4% disagreed or strongly disagreed with “I have never been interested in the Melbourne Cup”). Few reported less interest in the Melbourne Cup due to concerns with gambling (7.14% agree or strongly agree). A little over a fifth reported (21.43%) less interest in the Melbourne Cup due to animal welfare concerns. Men were over-represented among Gamblers (χ^2^ = 22.043, df = 1, p-value <0.001) and were younger (-0.95, SE = 0.19, p <0.01) than the Devotees.

**“Paradoxical-voters”** This cluster included 54 (5.3%) respondents. Paradoxical-voters provided contradictory responses throughout the survey, with a majority all agreeing or strongly agreeing with all six statements, despite the contradictions of doing so. Paradoxical-voters were overrepresented by males (χ^2^ = 10.311, df = 1, p-value = 0.001) and were younger than Devotees (-1.78, SE = 0.26, p <0.01)

## 4. Discussion

There are two main limitations to this study. First, the representativeness of the sample is limited by the convenience sampling strategy. However, it would not be unreasonable to assume that respondents had basic levels of English and online literacy as well as sufficient interest in the topics of gambling, racing and animal welfare to engage in the poll. Moreover, the polling company has a legitimate presence in Australia. Responses are made available weekly to online subscribers and a report is published in The Guardian Australia newspaper.

Second, the validity of the data is limited by some presumptuous wording of the survey statements. Whilst data were provided by a reputable independent research company, they were collected for a different aim than that discussed in this study. The six statements to which respondents indicated their agreement, disagreement and unsureness were designed to provide high rates of completion. For the purposes of this study, the validity of the statements may have been lowered by their inclusion of a frequency in the question form or a presumed relationship between two variables.

For example, Statement 1 (“I regularly bet on horse races”) would most likely provide data with higher validity around betting frequency if it had collected numerical data around the number of occasions during which someone had bet over a stated period of time. Statement 2 (“I rarely bet on horse races but will be watching the Melbourne Cup and placing a bet”) would most likely provide data with a more valid reflection of the prevalence of those whose betting on the Melbourne Cup is atypical of their general betting behavior if it simply asked about the intention to watch the Cup and place a bet, and was compared with data from Statement 1. Moreover, the inclusion of ‘watch’ and ‘place a bet’ may have yielded different data to a question asking only about ‘watching’ or ‘only about betting’. The separation of Statement 2 into those two variables would then have provided more valid data sought from Statement 3 (“I will watch the Melbourne Cup but will not place a bet”).

Statements 4, 5 and 6 were about interest in the Cup but were limited to statements about never being interested (Statement 4) or about becoming less interested due to a) concerns with gambling (Statement 5) and b) concerns with animal cruelty (Statement 6). Certainly, attitudes towards gambling and animal cruelty are mutually inclusive in animal-based gambling [33, 34]. Nonetheless, validity was lowered by Statement 4 not providing an ordinal scale for level of interest and Statements 5 and 6 providing two pre-specified reasons for lowered interest.

The caveat in Statement 5 around gambling did not specify ‘problem gambling’, hence it is unclear what kind of gambling was most likely to be under consideration when responses were provided by respondents.

The caveat in Statement 6 around animal cruelty concerns may account for over or under-emphasis on gambling or animal cruelty depending on how a participant prioritized the reasons for their declining interest compared to their declining interest which may have been for other reasons (such as boredom, politics, concerns with alcohol, reduced income, etc.). In particular, not all those who are against animal cruelty perceive horse racing as cruel [35]. How such people in our sample responded to the social desirability bias of not wanting to appear to tolerate animal cruelty versus any strong convictions that racing is not cruel, or resolved the cognitive dissonance [36] of being interested in–or betting on a sport that others consider cruel remains to be determined. Cognitive dissonance may even be particularly salient in this context given that human society is fraught with contradictory relationships to animals [37] and views range across spectrum from (at least) welfare to rights [38]. Finally, response to Statement 6 may have been different had the less provocative term ‘welfare’ been used instead of ‘cruelty’.

The limitations imposed on the responses that respondents were able to provide should be taken into consideration in the interpretation of the data presented here. Moreover, our findings and presentation of clusters are not exhaustive. There are other perspectives and clusters in the sample and general population which are beyond the scope of this paper. However, the aim of this study was not to discuss data in positivistic terms of representativeness and statistical significance. That would be disingenuous given the aforementioned limitations in sampling and design. Rather, the aim of this study was to conduct a preliminary exploration of associations between demographic variables and attitudes, as well as to initiate a non-binary understanding of attitudes towards the Melbourne Cup, gambling and animal cruelty.

This study suggests that attitudes towards the Melbourne Cup varied among the Australian population and are much more complex that simple binary views of being for or against Thoroughbred horse racing, gambling or animal cruelty. Therefore, despite being collected outside of academia, the data provide an opportunity to consider an important question that otherwise might be difficult to attract funding support, given corporate and nationalistic interests.

In particular, data also illustrate how stated behaviours and opinions vary demographically, especially in relation to gender, employment status and age. Contextualising findings within the literature is problematic, given that most of the research on gambling relates to specific populations, problematic or pathological gambling, online technologies and risk taking and sensation seeking behaviours, and is somewhat dated [39]. Intra-data comparisons do, however, yield some interesting findings.

Our results revealed that men showed more agreement with Statement 1 (“I regularly bet on horse races”), thus identifying themselves as regular gamblers on horse-races. In fact, 76% of those who agreed and strongly agreed with this statement were male. However, there was no association between gender and Statement 2, with 35.6% female respondents, and a similar 38.2% of male respondents agreeing or strongly agreeing, that despite not regularly gambling on horse-racing, they intended to watch the Melbourne Cup and place a bet. These findings suggest that betting behavior around Australia’s most iconic horse race is atypical from racehorse gambling behavior throughout the year and that the novelty of betting on the Melbourne Cup is salient to men and women alike.

Some gendered differences were identified in relation to reported losses of interest in the Melbourne Cup due to concerns for animal cruelty (Statement 6), which was higher amongst female respondents. This is consistent with a general trend that women tend to show more concern for animal welfare than men [40], although across research on this subject there appears to be more variation within than between gender categories [41].

Despite no consistent relationship between household income and intention to place a bet on the Melbourne Cup (inferred from Statement 2), there was an association between full- or part-time employment and intention to place a bet on the Melbourne Cup. While Melbourne Cup day is a public holiday in Victoria, it is not in the rest of Australia, so this association may be due to either formal or informal office sweepstakes or other occupational social pressures to gamble.

Setting aside the Gamblers and the Paradoxical-voters, the remaining cluster showing the greatest intention of watching the Melbourne Cup and gambling on it are the Devotees, almost two thirds of whom agreed or strongly agreed they would watch the race and place a bet. Few of these Devotees report having either gambling or animal welfare concerns that interfere with their interest in the Cup, fewer even than the Gamblers cluster. It may be this group which is engaging with the Melbourne Cup as an iconic event, such that placing a bet is a part of fully participating in the ritual, and this might explain the unexpectedly even gender ratio (roughly 40% female to 60% male–or 51% female to 49% male if grouped with the Gamblers cluster) among this cluster.

Aligning with reports of high gambling rates among younger people than older people [42]), we found fewer people over 65 years in our Gamblers cluster than expected under a condition of no association between age and group, but more people over 65 years than expected amongst Devotees, fewer than expected among the Flaneurs but more among the Casuals. Indeed, the over 65 years group was one of two age groups with somewhat different from expected cluster distributions, with the other group being the 25–29 years group in which Gamblers were overrepresented and Casuals were somewhat underrepresented.

There are some indications in this study that interest in the Melbourne Cup is stronger for older age brackets than younger ones. Younger people were more likely to indicate that they had never been interested in the Melbourne Cup, and the Disapprover and the Flaneur clusters were both significantly younger than Devotees. The Paradoxical-voting cluster tended to be younger rather than older people and were more likely to be male.

Finally, with specific regard to gambling behavior, the poll did not differentiate between different forms of gambling. Research suggests that the new mode of internet gamblers differ in many ways from existing pre-gamblers [43]. They may also have different perceptions of animal cruelty and the welfare of Thoroughbred racehorses than offline gamblers.

## 5. Conclusions

Australia’s most iconic horse race is also one of the most contentious events in Australia’s public arena. The aims of this study were to discern relationships between the stated attitudes and behaviours of survey respondents and their demographic attributes, and to explore how attitudes toward the Melbourne Cup intersect with concerns about animal welfare and gambling.

Some associations were found between stated behaviours and demographics in relation to gender, employment status and age. Men were more likely to regularly bet on horse races, people with full or part-time employment were more likely to intend to place a bet on the Melbourne Cup and women were more likely to report lessening interest in the Melbourne Cup due to concerns for animal cruelty. Intentions to place a bet appeared to be unaffected be gender or income.

Six clusters were identified. Devotees (31%) were unlikely to identify as gamblers but were very interested in watching and betting on the Cup, showing consistency over time. Flaneurs (22%) were neither interested in betting in general, nor the Melbourne Cup in particular. Disapprovers (16%) were not regular gamblers and were unlikely to watch and/or place a bet on the Cup. They reflected dissenters who had never approved of the Melbourne Cup race as well as apostates who had lost interest and reported changing their behaviours over time. Casuals (14%) never bet on horse races but were very interested in watching the Melbourne Cup horse race. Gamblers (12%) were those for whom the Melbourne Cup was probably just another horse race they regularly bet on. Lastly, Paradoxical-voters (5%) were those who completed the survey but selected the first response available to them.

Devotees and Gamblers are the most enthusiastic gamblers on the Melbourne Cup, but at only 43%, they are outweighed by the disinterested Flaneurs, Disapprovers and Casuals who are unlikely to place a bet (52%). Still, the novelty of the Melbourne Cup seemed to inspire 31% of those who would not identify as gamblers to place a bet. If the future of Australia’s Melbourne Cup horse race is dependent on the support of punters, findings suggest that whilst support seems solid, it may also be noncommittal and vulnerable to change. Indeed, this vulnerability could account for the 2019 Melbourne Cup experiencing a 24 year record low in attendance following the airing of a damning television documentary about the industry’s inability to track levels of ‘wastage’ or ensure animal welfare standards in abattoirs and slaughter houses [24]. As this study is based on data collected prior to the documentary, findings provide a foundation for future comparative research into the strength of punter commitment, vulnerability to negative press and the implications for the social license to race and gamble on horses.

## Supporting information

S1 File (PDF)

S1 Table Demographic data from n = 1028 survey respondents indicating their agreement with six attitudes regarding the annual Melbourne Cup Thoroughbred horse race. n = number of respondents selecting this demographic option, % percentage of respondents selecting this option. *Italicised responses* indicate respondent declined to answer and are not included in percentage calculations.
(DOCX)
