# Supplementary material for: The race that segments a nation: Findings from a convenience poll of attitudes toward the Melbourne Cup Thoroughbred horse race, gambling and animal cruelty
Source: PLoS One. 2021 Mar 24;16(3):e0248945. doi: 10.1371/journal.pone.0248945 (PMC7990293; doi:10.1371/journal.pone.0248945)
Supplement: S1 File — (PDF) [file pone.0248945.s001.pdf]

PLOS ONE

The race that segments a nation: Findings from a convenience poll of attitudes toward the Melbourne Cup Thoroughbred horse race, gambling and animal cruelty

--Manuscript Draft--

**Manuscript Number:**	PONE-D-20-21090R1
**Article Type:**	Research Article
**Full Title:**	The race that segments a nation: Findings from a convenience poll of attitudes toward the Melbourne Cup Thoroughbred horse race, gambling and animal cruelty
**Short Title:**	Findings from a convenience poll of attitudes toward the Melbourne Cup
**Corresponding Author:**	Bethany Jessica Wilson, BVSc<br>University of Sydney<br>Sydney, NSW AUSTRALIA
**Keywords:**	Horse Racing; Equine welfare; Animal Welfare; Gambling
**Abstract:**	The annual Melbourne Cup Thoroughbred horse race has iconic status among many Australians but sits in the context of increasing criticism of the welfare of Thoroughbred racing horses and the ethics of gambling. Despite heated debates and protests playing out in the public domain, there is scant empirical research to document Australian attitudes to the Melbourne Cup, or horse racing more generally Specifically, little is known about how support for or against the Melbourne Cup correlate with age, gender, income and level of education. To provide a more nuanced understanding of attitudes towards the cup beyond the rudimentary binaries of those who are 'for' or 'against' gambling and horse racing, the purpose of the study was to identify clusters of people with particular views. An opportunistic survey collected data on respondents' gender, age, place of residence, weekly income, employment status and highest level of education, and sought their level of agreement with six statements about the Melbourne Cup, gambling and animal cruelty. Ordinal logistic regression and Chi-square analysis were used to evaluate the age and gender of respondents in clusters respectively. Agreement with the statements revealed some significant associations. Male respondents were at greater odds for agreement with the statement: I regularly bet on horse races (OR= 2.39; 95% CI=1.78-3.22) as were respondents aged 18-19 years (OR= 2.88; 95% CI=1.13-7.35) and 20-24 years (OR= 1.90; 95% CI 1.00-3.62) compared with the median 35-40 years age bracket. Agreement with the statement: I will watch the Melbourne Cup but will not place a bet was more likely among the full-time employed (OR= 1.60; 95% CI=1.10- 2.32), for those aged 20-24 years (OR=1.85; 95% CI=1.16-2.95). The odds of increasing agreement with the statement: I have never been interested in the Melbourne Cup were multiplied by 0.87 (95% CI=0.82-0.92) with each successive five-year age bracket. The most useful of the predictor variables for agreement was level of education. The odds of increasing with the statement: I have become less interested in the Melbourne Cup over recent years because of my concerns with gambling were multiplied by 1.09 (95% CI=1.02-1.15) for each increased level of education. Agreement with the statement: I have become less interested in the Melbourne Cup because of my concerns about animal cruelty was weaker amongst male respondents (OR= 0.62; 95% CI=0.48-0.80), and those in increasing age brackets (OR= 0.88; 95% CI=0.83-0.93). A series of six clusters were identified that show how certain attributes of respondents characterise their responses. The authors labelled these clusters "Devotees" (n=313; 30.4% of respondents), "Flaneurs" (n=244; 21.8% of respondents), "Disapprovers" (n=163; 15.9% of respondents), "Casuals" (n=148; 14.4% of respondents), "Gamblers" (n=126; 12.3% of respondents) and "Paradoxical-voters" (n=54; 5.3% of respondents). The implications for support of the Melbourne Cup are explored.
**Order of Authors:**	Bethany Jessica Wilson, BVSc
	Kirrilly R. Thompson
	Paul Damien McGreevy
**Opposed Reviewers:**	
**Response to Reviewers:**	Response to Reviewers

Powered by Editorial Manager® and ProduXion Manager® from Aries Systems Corporation

The authors would like to very much thank the Reviewers and Academic Editors for their very helpful suggestions regarding this manuscript. Please see our detailed response and a record of the changes made to the manuscript below.

1. Please ensure that your manuscript meets PLOS ONE's style requirements, including those for file naming. The PLOS ONE style templates can be found at https://journals.plos.org/plosone/s/file?id=wjVg/PLOSOne_formatting_sample_main_body.pdf and https://journals.plos.org/plosone/s/file?id=ba62/PLOSOne_formatting_sample_title_authors_affiliations.pdf

Following this guideline
•The simple summary has been removed
•Major Sections have been headed with Size 18 Bold
•Italic Face has been removed from Minor section headings and changed to Bold Size 16
•Figure citations have been changed to “Fig. 1” and “Fig. 2”.
•Figure Titles have been changed to Bold Face\
•Level 3 Headings have been changed to Bold Face Size 14
•Headings changed to sentence case
•Double spacing added
•Article Title Unbolded
•Pilcrows added to author names

2. In your Methods section, please provide additional information about the participant recruitment method and the demographic details of your participants. Please ensure you have provided sufficient details to replicate the analyses such as: a) the recruitment date range (month and year), b) a description of any inclusion/exclusion criteria that were applied to participant recruitment, c) a table of relevant demographic details, d) a statement as to whether your sample can be considered representative of a larger population, e) a description of how participants were recruited, and f) descriptions of where participants were recruited and where the research took place.

a)This has been added

“The fortnight prior to 6th November 2018”

b) The recruitment process is in commercial confidence, however all Essential Research staff hold Australian Market and Social Research Society (AMSRS) membership and are bound by professional codes of behaviour.

c) This table has been prepared (Supplementary Table 1)

d)The following statement has been added.

“While the process is intended to sample a random sample of the population, sampling errors due to lack of 100% response rate of invited respondents and gaps in coverage of the original pool from which invited respondents were sourced cannot be ruled out.”

e) f) While the exact process is a matter of commercial confidence, it is understood that approximately 7000-8000 participants from a larger (~100,000) Australia-wide panel are randomly invited to participate in each omnibus online interview, resulting in a variable response rate which, in this case, was 1028. As stated, the questions were presented online, and the residential details of respondents are now included in Supplementary Table 1.

3. We note that you have stated that you will provide repository information for your data at acceptance. Should your manuscript be accepted for publication, we will hold it until you provide the relevant accession numbers or DOIs necessary to access your

Powered by Editorial Manager® and ProduXion Manager® from Aries Systems Corporation

data. If you wish to make changes to your Data Availability statement, please describe these changes in your cover letter and we will update your Data Availability statement to reflect the information you provide.

Contrary to our initial hopes, we have not obtained permission to release the data, although we encourage, in good faith, other researchers to apply to Essential Media for access to this data as we did.

We have updated our cover letter accordingly to reflect this information. We kindly request that our Data Availability statement be amended as such.

4. PLOS requires an ORCID iD for the corresponding author in Editorial Manager on papers submitted after December 6th, 2016. Please ensure that you have an ORCID iD and that it is validated in Editorial Manager. To do this, go to 'Update my Information' (in the upper left-hand corner of the main menu), and click on the Fetch/Validate link next to the ORCID field. This will take you to the ORCID site and allow you to create a new iD or authenticate a pre-existing iD in Editorial Manager. Please see the following video for instructions on linking an ORCID iD to your Editorial Manager account: https://www.youtube.com/watch?v=_xcclfuvtxQ

This step has been completed.

Reviewers' comments:
1)Reviewer 2 expresses concern about the "strength" of statements "animal cruelty" (Simple summary) And "significant welfare costs for horses and people" Although they later state that these are addressed in the discussion.
The removal of the simple summary in accordance with the journal requirements has addressed this first concern. The second statement has been moderated by the inclusion of the word "potentially"

"But despite its economic and social benefits, Thoroughbred racing in general, and the Melbourne Cup day in particular, potentially carry significant welfare costs to both horses and people."

As the reviewer notes, further discussion is to be found later in the manuscript.

2)Reviewer 2 would like the following paper to be referenced
https://www.mdpi.com/2071-1050/12/5/1706/htm
Heleski, C.; Stowe, C.J.; Fiedler, J.; Peterson, M.L.; Brady, C.; Wickens, C.; MacLeod, J.N. Thoroughbred Racehorse Welfare through the Lens of 'Social License to Operate—With an Emphasis on a U.S. Perspective. Sustainability 2020, 12, 1706.

This reference has been added (new Reference 14)

Two additional new references (new Reference 12, and new Reference 13) have also been added.

3)The reviewer would like us to clarify that this data was collected prior to a major public expose in the materials and methods

The following text has been added to Materials and Methods.

"We note that these data were collected well before the profile of Australian Racing was challenged by documentaries such as The Final Race (ABC TV's 7.30 Report, 17th October 2019)."

4)Reviewer would like options 5 and 6 from the list to align (I believe geometrically!) with 1 through 4 (Page 9)

Powered by Editorial Manager® and ProduXion Manager® from Aries Systems Corporation

	This has been done.<br><br>5)The Reviewer notes that there is a word missing from “The association between respondents and were explored by ordinal logistic regression”<br><br>The words “respondent demographics” have been inserted as follows.<br><br>“The association between respondents and respondent demographics were explored by ordinal logistic regression using the polr function of the MASS package in R [28,29].”<br><br>6)The Reviewer would like a clarification for the reader on the way that the sums total in Table 1 on Page 10. They suggest adding language like “total rows sum to 100% horizontally” and “each sub category, divided by gender, will sum vertically”.<br><br>To address this point the reviewers’ suggested text has been added to the table legend and the total rows have been bolded to distinguish them visually.<br><br>7)The Reviewer is uncertain of the validity and the utility of the median column in Table 1 and suggests deleting it.<br><br>This change has been made.<br><br>8)The Reviewer notes an extra “this” in the statement 5 text on Page 11 “When education was modelled ordinally, the odds of increasing agreement with this this statement were multiplied by 1.09 (95% CI=1.02-1.15) for each increased level of education.”<br><br>The extra ‘this’ has been deleted. Thank you.<br><br>9)The Reviewer would like “in the public arena” to be changed to “in Australia’s public arena” Page 15. First sentence of Conclusion.<br><br>This change has been made.
**Additional Information:**	
**Question**	**Response**
**Financial Disclosure**<br><br>Enter a financial disclosure statement that describes the sources of funding for the work included in this submission. Review the submission guidelines for detailed requirements. View published research articles from *PLOS ONE* for specific examples.<br><br>This statement is required for submission and **will appear in the published article** if the submission is accepted. Please make sure it is accurate.	Funding for this study was received from RSPCA Australia. BW https://www.rspca.org.au/ The funders had no role in study design, data collection and analysis, decision to publish, or preparation of the manuscript. Additional funding was provided by the Sydney School of Veterinary Science.

Powered by Editorial Manager® and ProduXion Manager® from Aries Systems Corporation

**Unfunded studies**<br>Enter: *The author(s) received no specific funding for this work.*<br><br>**Funded studies**<br>Enter a statement with the following details:<br>• Initials of the authors who received each award<br>• Grant numbers awarded to each author<br>• The full name of each funder<br>• URL of each funder website<br>• Did the sponsors or funders play any role in the study design, data collection and analysis, decision to publish, or preparation of the manuscript?<br>• **NO** - Include this sentence at the end of your statement: *The funders had no role in study design, data collection and analysis, decision to publish, or preparation of the manuscript.*<br>• **YES** - Specify the role(s) played.<br><br>* typeset	
**Competing Interests**<br><br>Use the instructions below to enter a competing interest statement for this submission. On behalf of all authors, disclose any competing interests that could be perceived to bias this work—acknowledging all financial support and any other relevant financial or non-financial competing interests.<br><br>This statement **will appear in the published article** if the submission is accepted. Please make sure it is accurate. View published research articles from *PLOS ONE* for specific examples.	The authors have declared that no competing interests exist.

**NO authors have competing interests**<br><br>Enter: *The authors have declared that no competing interests exist.*<br><br>**Authors with competing interests**<br><br>Enter competing interest details beginning with this statement:<br><br>*I have read the journal's policy and the authors of this manuscript have the following competing interests: [insert competing interests here]*<br><br>* typeset	
**Ethics Statement**<br><br>Enter an ethics statement for this submission. This statement is required if the study involved:<br><br>• Human participants<br>• Human specimens or tissue<br>• Vertebrate animals or cephalopods<br>• Vertebrate embryos or tissues<br>• Field research<br><br>Write "N/A" if the submission does not require an ethics statement.<br><br>General guidance is provided below. Consult the submission guidelines for detailed instructions. **Make sure that all information entered here is included in the Methods section of the manuscript.**	N/A

**Format for specific study types**<br><br>**Human Subject Research (involving human participants and/or tissue)**<br>• Give the name of the institutional review board or ethics committee that approved the study<br>• Include the approval number and/or a statement indicating approval of this research<br>• Indicate the form of consent obtained (written/oral) or the reason that consent was not obtained (e.g. the data were analyzed anonymously)<br><br>**Animal Research (involving vertebrate animals, embryos or tissues)**<br>• Provide the name of the Institutional Animal Care and Use Committee (IACUC) or other relevant ethics board that reviewed the study protocol, and indicate whether they approved this research or granted a formal waiver of ethical approval<br>• Include an approval number if one was obtained<br>• If the study involved *non-human primates*, add *additional details* about animal welfare and steps taken to ameliorate suffering<br>• If anesthesia, euthanasia, or any kind of animal sacrifice is part of the study, include briefly which substances and/or methods were applied<br><br>**Field Research**<br><br>Include the following details if this study involves the collection of plant, animal, or other materials from a natural setting:<br>• Field permit number<br>• Name of the institution or relevant body that granted permission	
**Data Availability**<br><br>Authors are required to make all data underlying the findings described fully available, without restriction, and from the time of publication. PLOS allows rare exceptions to address legal and ethical concerns. See the PLOS Data Policy and FAQ for detailed information.	No - some restrictions will apply

A Data Availability Statement describing where the data can be found is required at submission. Your answers to this question constitute the Data Availability Statement and **will be published in the article**, if accepted.<br><br>**Important:** Stating 'data available on request from the author' is not sufficient. If your data are only available upon request, select 'No' for the first question and explain your exceptional situation in the text box.<br><br>Do the authors confirm that all data underlying the findings described in their manuscript are fully available without restriction?	
**Describe where the data may be found in full sentences. If you are copying our sample text, replace any instances of XXX with the appropriate details.**<br><br>• If the data are **held or will be held in a public repository**, include URLs, accession numbers or DOIs. If this information will only be available after acceptance, indicate this by ticking the box below. For example: *All XXX files are available from the XXX database (accession number(s) XXX, XXX.).*<br>• If the data are all contained **within the manuscript and/or Supporting Information files**, enter the following: *All relevant data are within the manuscript and its Supporting Information files.*<br>• If neither of these applies but you are able to provide **details of access elsewhere**, with or without limitations, please do so. For example:<br><br>*Data cannot be shared publicly because of [XXX]. Data are available from the XXX Institutional Data Access / Ethics Committee (contact via XXX) for researchers who meet the criteria for access to confidential data.*<br><br>*The data underlying the results presented in the study are available from (include the name of the third party*	Data may be obtained by contacting Essential Media via https://essentialmedia.com.au/contact-us. The authors had no special access to this data.

and contact information or URL).<br>• This text is appropriate if the data are owned by a third party and authors do not have permission to share the data.<br><br>* typeset	
Additional data availability information:	

THE UNIVERSITY OF SYDNEY

Dr Bethany Wilson
Honorary Affiliate

16th December 2020

Professor Chris Rogers
Academic Editor
PLOS ONE

Re: The race that segments a nation: Findings from a convenience poll of attitudes toward the Melbourne Cup Thoroughbred horse race, gambling and animal cruelty

Dear Professor Rogers,

Please find the accompanying original research article that explores Australian attitudes toward the Melbourne Cup, gambling and animal cruelty. Our manuscript has been edited according to the requests of the Reviewer 2 and Academic Editor. Please see the included Response to Reviewers for details.

Like our previous article on whipping (McGreevy et al 2018), it uses findings from a convenience poll. It analyses perspectives on a major horse race and Australian national icon and reveal novel information about attitudes towards horse racing among the general public and may help contribute to the current ongoing national and international conversations about horse racing welfare.

We have unfortunately have not secured permission from the commercial polling company to release the full data set, however we encourage, in good faith, for other interested reviewers to apply to *Essential Media* for access to this data, as we did. We kindly request that our Data Availability statement be amended, as such. **Data may be obtained by contacting Essential Media via https://essentialmedia.com.au/contact-us. The authors had no special access to this data.**

Additionally we request that our Funding statement be updated to

Funding for this study was received from RSPCA Australia. BW https://www.rspca.org.au/ The funders had no role in study design, data collection and analysis, decision to publish, or preparation of the manuscript. **Additional funding was provided by the Sydney School of Veterinary Science.**

Faculty of Veterinary Science
Room 206, R.M.C. Gunn Building (B19)
University of Sydney
NSW 2006 Australia

T +61 2 9351 2810
F +61 2 9351 3957
E bethany.wilson@sydney.edu.au

ABN 15 211 513 464
CRICOS 00026A

THE UNIVERSITY OF SYDNEY

Yours sincerely,

Bethany Wilson on behalf of the authors

Reference McGreevy, P.D., Griffiths, M.D., Ascione, F.R., Wilson, B. 2018. Flogging tired horses: Who wants whipping and who would walk away if whipping horses were withheld*? PLoS ONE. 13(2):e0192843.*

Manuscript Click here to access/download;Manuscript;Manuscript_B.docx

The race that segments a nation: Findings from a convenience poll of attitudes toward the Melbourne Cup Thoroughbred horse race, gambling and animal cruelty

Bethany J. Wilson[1¶*], Kirrilly R. Thompson[2¶] and Paul D. McGreevy [1¶]

[1] Sydney School of Veterinary Science, Faculty of Science, University of Sydney, Sydney, NSW 2006, Australia;

[2] University of South Australia Business School 61 North Terrace, Adelaide SA 5000

* Correspondence: bethany.wilson@sydney.edu.au;

¶ These authors contributed equally to this work

Abstract

The annual Melbourne Cup Thoroughbred horse race has iconic status among many Australians but sits in the context of increasing criticism of the welfare of Thoroughbred racing horses and the ethics of gambling. Despite heated debates and protests playing out in the public domain, there is scant empirical research to document Australian attitudes to the Melbourne Cup, or horse racing more generally . Specifically, little is known about how support for or against the Melbourne Cup correlate with age, gender, income and level of education. To provide a more nuanced understanding of

attitudes towards the cup beyond the rudimentary binaries of those who are 'for' or 'against' gambling and horse racing, the purpose of the study was to identify clusters of people with particular views. An opportunistic survey collected data on respondents' gender, age, place of residence, weekly income, employment status and highest level of education, and sought their level of agreement with six statements about the Melbourne Cup, gambling and animal cruelty. Ordinal logistic regression and Chi-square analysis were used to evaluate the age and gender of respondents in clusters respectively. Agreement with the statements revealed some significant associations. Male respondents were at greater odds for agreement with the statement: *I regularly bet on horse races* (OR= 2.39; 95% CI=1.78-3.22) as were respondents aged 18-19 years (OR= 2.88; 95% CI=1.13-7.35) and 20-24 years (OR= 1.90; 95% CI 1.00-3.62) compared with the median 35-40 years age bracket. Agreement with the statement: I will watch the Melbourne Cup but will not place a bet was more likely among the full-time employed (OR= 1.60; 95% CI=1.10- 2.32), for those aged 20-24 years (OR=1.85; 95% CI=1.16-2.95). The odds of increasing agreement with the statement: *I have never been interested in the Melbourne Cup* were multiplied by 0.87 (95% CI=0.82-0.92) with each successive five-year age bracket. The most useful of the predictor variables for agreement was level of education. The odds of increasing with the statement: *I have become less interested in the Melbourne Cup over recent years because of my concerns with gambling* were multiplied by 1.09 (95% CI=1.02-1.15) for each increased level of education. Agreement with the statement: *I have become less interested in the Melbourne Cup because of my concerns about animal cruelty* was weaker amongst male respondents (OR= 0.62; 95% CI=0.48-0.80), and those in increasing age brackets (OR= 0.88; 95% CI=0.83-0.93). A series of six clusters were identified that show how certain attributes of respondents characterise their responses. The authors labelled these clusters "Devotees" (n=313; 30.4% of respondents), "Flaneurs" (n=244; 21.8% of respondents), "Disapprovers" (n=163; 15.9% of respondents), "Casuals" (n=148; 14.4% of respondents), "Gamblers" (n=126; 12.3% of respondents) and "Paradoxical-voters" (n=54; 5.3% of respondents). The implications for support of the Melbourne Cup are explored.

Keywords: horse-racing; gambling; animal welfare;

1.Introduction

The Melbourne Cup is a Thoroughbred horse race which takes place on the first Tuesday of November every year in the Melbourne suburb of Flemington, as the premier event of the Melbourne Spring Carnival [1]. First run in 1861, the race has become both a prominent part of the Australian national culture, listed with barbeques, football and ANZAC day as a core cultural symbol of Australian identity [2] and also a significant event on the global racing calendar, comparable to the Grand National, Kentucky Derby and Japan Cup [3,4].

Growing from the estimated crowd of four thousand who attended the first Melbourne Cup day [2] to turn-outs well in excess of 100,000 in the modern era [1,4], the event contributes an estimated AUD350 million to the state economy [4]. Annual betting of more than AUD105 million on this single race has been recorded, and global television audiences have been estimated at more than 1 billion [4]. In addition to gambling and the sport of horse-racing itself, since the 1960s the Melbourne Cup has also become intimately associated with fashion, and celebrity culture, creating another face of horse-racing with which the public can engage [2,5].

But despite its economic and social benefits, Thoroughbred racing in general[6], and the Melbourne Cup day in particular, potentially carry significant welfare costs to both horses and people. In addition to high profile deaths, such as the euthanasia of racehorse Cliffsofmoher following an injury early in the 2018 Melbourne Cup and the sudden death of Admire Rakti shortly after racing in 2014, In addition to the high profile deaths of Melbourne Cup runners on track or shortly after (7

horses since 2013), Thoroughbred racing is associated with widespread wastage[5] and acute and chronic pain from musculoskeletal injuries [7], pulmonary haemorrhages [8], gastric ulcers [9] and increasing public distaste for the use and consequence of equipment such aswhips [10-12] and tongue-ties [13]. Additionally, problem gambling is a widespread financial and mental health issue among Australians [14]. These concerns can have significant implications for the Thoroughbred racing industry's social license to operate [15,16] . Horse racing has been increasingly controversial in Australia over the past decades, mostly in relation to whip use [17-20] and injury and fatality rates in jumps racing [21-23]. More recently, there was a shared outcry from racing proponents and opponents alike in response to a *7.30 Report* exposé into the end of life for horse 'wastage' from the Australian Thoroughbred racing industry, particularly horses which had raced in New South Wales [24].

The ethical use of horses demands that we consider the welfare impact that horse-racing has on horses despite the economic and social benefits of horse-racing [25-27]. The Melbourne Cup, despite (or perhaps because of) its status as a cultural icon [28], is no exception.

Ahead of the 2018 Melbourne Cup, a commercial poll of adult Australians revealed that, when asked about horse-racing in general, 8% professed high interest in the sport, 20% reported moderate interest, while 70% said they had low or no interest [29]. However, although only 19% of the sample reported they regularly bet on horse-races, 38% said they would be watching the Melbourne Cup that year and would place a bet. Furthermore, 33% said they would be watching the event but not placing a bet. These data seem to confirm the iconic status that the Melbourne Cup has for many Australians [29].

Following the example offered by an earlier report on the use of polling data from a third party [20], the current study returns to the original data to explore relationships among these attitudes and

respondents' income, employment status, age and sex. It also explores how attitudes toward the Melbourne Cup intersect with concerns about animal welfare concerns and problem gambling.

2. Materials and methods

2.1 Data

The data collection was performed by Essential Research, a division of Essential media, who, in addition to demographic data about gender, age, place of residence, weekly income, employment status and highest level of education, electronically polled respondents for their level of agreement with the following six statements:

1. I regularly bet on horse races
2. I rarely bet on horse races but will be watching the Melbourne Cup and placing a bet
3. I will watch the Melbourne Cup but will not place a bet
4. I have never been interested in the Melbourne Cup
5. I have become less interested in the Melbourne Cup over recent years because of my concerns with gambling
6. I have become less interested in the Melbourne Cup because of my concerns about animal cruelty

The questions were asked online as part of a larger omnibus of questions on a variety of topics allowing several days for survey completion under the supervisions of members of the Australian Market and Social Research Society (AMSRS), acting under a professional code of behavior. This was undertaken in the fortnight prior to 6th November 2018. We note that these data were collected well before the profile of Australian Racing was challenged by documentaries such as The Final Race (ABC

TV's 7.30 Report, 17th of October 2019). While the process is intended to sample a random sample of the population, sampling errors due to lack of 100% response rate of invited respondents and gaps in coverage of the original pool from which invited respondents were sourced cannot be ruled out.

2.2.Analysis

2.2.1. Demographics

The association between respondents and respondent demographics were explored by ordinal logistic regression using the polr function of the MASS package in R [30,31].

The model used was

$Score_{ij} \sim Gender_i + Age_i + Residence_i + Income_i + Employment_i + Education_i$

Where $Score_{ij}$ = the Agreement Score (ie Strongly Disagree< Disagree<Don't know<Agree< Strongly Agree) of Participant i to statement j (where j is one of statements 1-6 above); $Gender_I$ = Whether Participant i identified as "Male" or "Female"; Age_i = the age in years bracket nominated by Participant i for themselves; $Residence_i$ = Civic area where Participant i said that they lived; $Income_i$= Participant i's nominated weekly income bracket; $Employment_i$ = Participant i's nominated employment status; $Education_i$ = Participant I's highest stated level of education.

2.2.2. Cluster analysis

A hierarchical cluster analysis was performed on the Agreement Scores of the six statements (using Gower distance) with the daisy and hclust functions [32]. "Don't know" was again placed centrally (i.e., Strongly Disagree< Disagree<Don't know<Agree< Strongly Agree).

Ordinal logistic regression and Chi-square analysis were used to evaluate the age and gender of respondents in clusters respectively.

3. Results

3.1. Demographics

A total of 1028 respondents completed the survey, of whom 526 (51.2%) were female and 502 (48.8%) were male. Their agreement with the six statements about the Melbourne Cup, gambling and horse racing were stratified by gender (See Table 1).

Table 1. Ordinal agreement among 1028 respondents with six statements regarding the Melbourne Cup and horse-racing stratified by gender.

Statement	Strongly Disagree	Disagree	Don't Know	Agree	Strongly Agree
S1: I regularly bet on horse races					
Female	348 (58%)	120 (56%)	16 (44%)	31 (28%)	11 (17%)
Male	255 (42%)	96 (44%)	20 (56%)	78 (72%)	53 (83%)
**Total**	**603 (59%)**	**216 (21%)**	**36 (4%)**	**109 (11%)**	**64 (6%)**
S2: I rarely bet on horse races but will be watching the Melbourne Cup and placing a bet					

Female	202 (54%)	98 (47%)	39 (54%)	119 (49%)	68 (51%)
Male	169 (46%)	111 (53%)	33 (46%)	123 (51%)	66 (49%)
**Total**	**371 (36%)**	**209 (20%)**	**72 (7%)**	**242 (24%)**	**134 (13%)**
S3: I will watch the Melbourne Cup but will not place a bet					
Female	189 (54%)	138 (52%)	47 (55%)	104 (45%)	48 (48%)
Male	158 (46%)	126 (48%)	39 (45%)	126 (55%)	53 (52%)
**Total**	**347 (34%)**	**264 (26%)**	**86 (8%)**	**230 (22%)**	**101 (10%)**
S4: I have never been interested in the Melbourne Cup					
Female	154	182	25	78	87
Male	139	159	27	108	69
**Total**	**293 (29%)**	**341 (33%)**	**52 (5%)**	**186 (18%)**	**156 (15%)**
S5: I have become less interested in the Melbourne Cup over recent years because of my concerns with gambling					
Female	187	179	40	67	53
Male	164	154	38	102	44
**Total**	**351 (34%)**	**333 (32%)**	**78 (8%)**	**169 (16%)**	**97 (9%)**
S6: I have become less interested in the Melbourne Cup because of my concerns about animal cruelty					
Female	148	164	41	85	88
Male	178	168	35	78	43
**Total**	**326 (32%)**	**332 (32%)**	**76 (7%)**	**163 (16%)**	**131 (13%)**

Frequency of response and (%) are offered. Total rows sum to 100% horizontally and each sub category, divided by gender, will sum vertically".

Statement 1: "I regularly bet on horse races". Male respondents were at greater odds than the average respondent for agreement with this statement (OR= 2.39; 95% CI=1.78-3.22), as were respondents aged 18-19 years (OR= 2.88; 95% CI=1.13-7.35) and 20-24 years (OR= 1.90; 95% CI 1.00-3.62) compared with the median 35-40 years age bracket, and income earners in the bracket AUD$1-$199 per week (OR= 3.07; 95% CI 1.26-7.47).

In contrast, female respondents, respondents earning in the range of AUD$1,250-$1,499 per week (OR=0.54; 95% CI=0.32-0.91) and students (OR= 0.35; 95% CI=0.16-0.79) were at lesser odds than average for agreement. Respondents over 64 years of age (OR= 0.34 95% CI=0.16-0.73) were in less agreement than the median 35-40 years age bracket. Testing for an interaction between Gender and Age was found it to be not significant (p=0.43).

Statement 2: "I rarely bet on horse races but will be watching the Melbourne Cup and placing a bet". Unlike the first statement about habitual gambling, male respondents were not significantly more likely to say that, although they rarely bet on horse-racing, they would bet on the Melbourne Cup (LR χ^2= 0.0382, df=1 p=0.85).

Those aged 20-24 years showed higher odds of agreement (OR=1.73; 95% CI=1.10-2.71) with this statement than average whereas those aged 30-34 (OR=0.64; 95% CI=0.44-0.94) showed lower agreement. Respondents in the AUD$1,250-$1,499 per week income range were at lower odds of intending to gamble on the Melbourne Cup, as they also were at lower odds of regular gambling (OR= 0.58; 95% CI=0.38-0.89). Those employed full- (OR=1.98; 95% CI=1.36- 2.89) and part-time (OR=1.49; 95% CI=1.01-2.20) were at increased odds compared to the average of agreement for this statement.

Statement 3: "I will watch the Melbourne Cup but will not place a bet". Agreement with this statement was more likely among the full-time employed (OR= 1.60; 95% CI=1.10- 2.32), for those aged 20-24 years (OR=1.85; 95% CI=1.16-2.95) and less likely for those aged 50-54 years (OR= 0.67; 95% CI=0.47-0.96).

Statement 4: "I have never been interested in the Melbourne Cup". The odds of agreement with this statement were highest among the relatively young age brackets 25-29 years (OR= 2.04; 95%

CI=1.40-2.97) and 30-34 (OR=1.61; 95% CI=1.10-2.35) and lowest in the older 60-64 years bracket (OR= 0.59; 95% CI=0.38- 0.92) compared to the average and in the 65 or older range 34 (OR=0.40; 95% CI=0.20-0.77) when compared to the 35-39 years range. This pattern is also true if age brackets are modelled ordinally with the odds of increasing agreement with the statement being multiplied by 0.87 (95% CI=0.82-0.92) with each successive five-year age bracket. Household income in the bracket AUD$600-$799 per week also significantly reduced the odds of agreement compared to average (OR=0.59; 95% CI=0.38-0.92).

Statement 5: "I have become less interested in the Melbourne Cup over recent years because of my concerns with gambling". The most useful of the predictor variables for this statement was level of education. When education was modelled ordinally, the odds of increasing agreement with this statement were multiplied by 1.09 (95% CI=1.02-1.15) for each increased level of education.

The odds of agreement were lowered for those in the income bracket of AUD$800-$999 per week. (OR= 0.55; 95% CI=0.36- 0.83).

Statement 6: "I have become less interested in the Melbourne Cup because of my concerns about animal cruelty". Male respondents (OR= 0.62; 95% CI=0.48-0.80), and increasing age brackets (OR= 0.88; 95% CI=0.83-0.93) were associated with lower odds of agreement with this statement as did the AUD$800-$999 per week household income bracket (OR=0.57; 95% CI=0.38-0.87).

3.2. Cluster analysis

Respondents were classified into six groups through agglomerative hierarchical clustering based on the Gower Distance. The hierarchical relationship between these six groups is shown by the dendrogram in Fig. 1.

Fig1: Cluster analysis of respondents (n=1028) by ordinal agreement with six statements regarding 1028 respondents' attitudes to the Melbourne Cup.

The demographics of the clusters are shown in Fig. 2.

Fig 2: Demographics of respondents (n=1028) assigned to six clusters by ordinal agreement with six statements regarding attitudes to the Melbourne Cup.

3.2.1. Clusters

The six clusters are described below in order from most to least represented within the sample.

"Devotees". This cluster included 313 (30.4%) respondents. These respondents did not report regular gambling on horse-races (99.7% disagree or strongly disagree with "I regularly bet on horses"). Nevertheless, they showed very high interest in the Melbourne Cup (99.4% disagreed or strongly disagreed with "I have never been interested in the Melbourne Cup") and many planned to bet on it (63.6% agreed or strongly agreed with "I rarely bet on horse races but will be watching the Melbourne Cup and placing a bet"). Very few of this group reported reduced interest in the Cup due to gambling or welfare concerns (99.4% disagreed or strongly disagreed with "I have become less interested in the Melbourne Cup over recent years because of my concerns with gambling "; 97.8% disagreed or strongly disagreed with "I have become less interested in the Melbourne Cup over

recent years because of my concerns about animal cruelty "). Women were over-represented among Devotees (χ^2 =7.2755, df = 1, p-value = 0.007).

"Flaneurs". This cluster included 224 (21.8%) respondents. Flaneurs did not report high rates of regular gambling on horse races (82.6% disagreed or strongly disagreed with "I regularly bet on horses") and they reported relatively low intention of watching the Melbourne Cup and placing a bet (16.1% agreed or strongly agreed with "I rarely bet on horse races but will be watching the Melbourne Cup and placing a bet". They showed relatively low interest in the Melbourne Cup (79 % agreed or strongly agreed with "I have never been interested in the Melbourne Cup", and only 3.6% disagreed or strongly disagreed). Few agreed or strongly agreed to having reduced interest in the Melbourne Cup due to concerns about gambling (6.7%), but more reported reduced interest due to animal welfare concerns (17.9%). Neither women nor men were significantly over-represented but respondents in this cluster were younger than Devotees (-0.70, SE=0.15, p<0.01).

"Disapprovers'". This cluster included 163 (15.9%) respondents. Disapprovers did not report regular gambling on horse races (98.2% disagreed or strongly disagreed with "I regularly bet on horses"). Less than a quarter of this group agreed that they were planning to watch the Cup, with (22.1%) or without betting (16.0%). Neither women nor men were significantly over-represented but respondents in this cluster were younger than Devotees (-0.49, SE=0.17, p<0.01). Some Disapprovers revealed apostatic views. They reported the greatest loss of interest in the Melbourne Cup due to moral and ethical concerns; 89.0% reported lessened interest due to concerns with gambling, and 74.2% due to concerns with animal cruelty. A reasonable number of respondents in this cluster revealed dissenting views, as 35.6% disagreed or strongly disagreed that they have never been interested in the Melbourne Cup.

"Casuals". This cluster included 148 (14.4%) respondents. Like the Devotees, these respondents did not report regular gamblers on horse races (100% disagree or strongly disagree with "I regularly bet on horses"). Nonetheless, they did show high interest in the Melbourne Cup (89.2 % disagreed or strongly disagreed with "I have never been interested in the Melbourne Cup"), but they do not generally plan to bet on it (86.5% agreed or strongly agreed with "I will watch the Melbourne Cup but will not place a bet"). About a third of these respondents reported reduced interest in the Cup due to concerns about animal welfare (33.8%) and slightly fewer due to concerns about gambling (31.1%). This cluster was not significantly older or younger than the Devotees and neither women nor men were overrepresented.

"Gamblers". This cluster included 126 (12.3%) respondents. Gamblers tended to report high levels of betting on horses in general (88.1% agree or strongly agree with "I regularly bet on horses"). They showed high interest in the Melbourne Cup (94.4% disagreed or strongly disagreed with "I have never been interested in the Melbourne Cup"). Few reported less interest in the Melbourne Cup due to concerns with gambling (7.14% agree or strongly agree). A little over a fifth reported (21.43%) less interest in the Melbourne Cup due to animal welfare concerns. Men were over-represented among Gamblers (χ^2=22.043, df=1, p-value <0.001) and were younger (-0.95, SE=0.19, p <0.01) than the Devotees.

"Paradoxical-voters" This cluster included 54 (5.3%) respondents. Paradoxical-voters provided contradictory responses throughout the survey, with a majority all agreeing or strongly agreeing with all six statements, despite the contradictions of doing so. Paradoxical-voters were overrepresented by males (χ^2 = 10.311, df = 1, p-value = 0.001) and were younger than Devotees (-1.78, SE=0.26, p <0.01)

4. Discussion

There are two main limitations to this study. First, the representativeness of the sample is limited by the convenience sampling strategy. However, it would not be unreasonable to assume that respondents had basic levels of English and online literacy as well as sufficient interest in the topics of gambling, racing and animal welfare to engage in the poll. Moreover, the polling company has a legitimate presence in Australia. Responses are made available weekly to online subscribers and a report is published in The Guardian Australia newspaper.

Second, the validity of the data is limited by some presumptuous wording of the survey statements. Whilst data were provided by a reputable independent research company, they were collected for a different aim than that discussed in this study. The six statements to which respondents indicated their agreement, disagreement and unsureness were designed to provide high rates of completion. For the purposes of this study, the validity of the statements may have been lowered by their inclusion of a frequency in the question form or a presumed relationship between two variables.

For example, Statement 1 ("I regularly bet on horse races") would most likely provide data with higher validity around betting frequency if it had collected numerical data around the number of occasions during which someone had bet over a stated period of time. Statement 2 ("I rarely bet on horse races but will be watching the Melbourne Cup and placing a bet") would most likely provide data with a more valid reflection of the prevalence of those whose betting on the Melbourne Cup is atypical of their general betting behavior if it simply asked about the intention to watch the Cup and place a bet, and was compared with data from Statement 1. Moreover, the inclusion of 'watch' and 'place a bet' may have yielded different data to a question asking only about 'watching' or 'only about betting'. The separation of Statement 2 into those two variables would then have provided more valid data sought from Statement 3 ("I will watch the Melbourne Cup but will not place a bet").

Statements 4, 5 and 6 were about interest in the Cup but were limited to statements about never being interested (Statement 4) or about becoming less interested due to a) concerns with gambling (Statement 5) and b) concerns with animal cruelty (Statement 6). Certainly, attitudes towards

gambling and animal cruelty are mutually inclusive in animal-based gambling [33,34]. Nonetheless, validity was lowered by Statement 4 not providing an ordinal scale for level of interest and Statements 5 and 6 providing two pre-specified reasons for lowered interest.

The caveat in Statement 5 around gambling did not specify 'problem gambling', hence it is unclear what kind of gambling was most likely to be under consideration when responses were provided by respondents.

The caveat in Statement 6 around animal cruelty concerns may account for over or under-emphasis on gambling or animal cruelty depending on how a participant prioritized the reasons for their declining interest compared to their declining interest which may have been for other reasons (such as boredom, politics, concerns with alcohol, reduced income, etc.). In particular, not all those who are against animal cruelty perceive horse racing as cruel [35]. How such people in our sample responded to the social desirability bias of not wanting to appear to tolerate animal cruelty versus any strong convictions that racing is not cruel, or resolved the cognitive dissonance [36] of being interested in – or betting on a sport that others consider cruel remains to be determined. Cognitive dissonance may even be particularly salient in this context given that human society is fraught with contradictory relationships to animals [37] and views range across spectrum from (at least) welfare to rights [38]. Finally, response to Statement 6 may have been different had the less provocative term 'welfare' been used instead of 'cruelty'.

The limitations imposed on the responses that respondents were able to provide should be taken into consideration in the interpretation of the data presented here. Moreover, our findings and presentation of clusters are not exhaustive. There are other perspectives and clusters in the sample and general population which are beyond the scope of this paper. However, the aim of this study was not to discuss data in positivistic terms of representativeness and statistical significance. That would be disingenuous given the aforementioned limitations in sampling and design. Rather, the aim of this study was to conduct a preliminary exploration of associations between demographic variables and

attitudes, as well as to initiate a non-binary understanding of attitudes towards the Melbourne Cup, gambling and animal cruelty.

This study suggests that attitudes towards the Melbourne Cup varied among the Australian population and are much more complex that simple binary views of being for or against Thoroughbred horse racing, gambling or animal cruelty. Therefore, despite being collected outside of academia, the data provide an opportunity to consider an important question that otherwise might be difficult to attract funding support, given corporate and nationalistic interests.

In particular, data also illustrate how stated behaviours and opinions vary demographically, especially in relation to gender, employment status and age. Contextualising findings within the literature is problematic, given that most of the research on gambling relates to specific populations, problematic or pathological gambling, online technologies and risk taking and sensation seeking behaviours, and is somewhat dated [39]. Intra-data comparisons do, however, yield some interesting findings.

Our results revealed that men showed more agreement with Statement 1 ("I regularly bet on horse races"), thus identifying themselves as regular gamblers on horse-races. In fact, 76% of those who agreed and strongly agreed with this statement were male. However, there was no association between gender and Statement 2, with 35.6% female respondents, and a similar 38.2% of male respondents agreeing or strongly agreeing, that despite not regularly gambling on horse-racing, they intended to watch the Melbourne Cup and place a bet. These findings suggest that betting behavior around Australia's most iconic horse race is atypical from racehorse gambling behavior throughout the year and that the novelty of betting on the Melbourne Cup is salient to men and women alike.

Some gendered differences were identified in relation to reported losses of interest in the Melbourne Cup due to concerns for animal cruelty (Statement 6), which was higher amongst female respondents. This is consistent with a general trend that women tend to show more concern for animal welfare than men [40], although across research on this subject there appears to be more variation within than between gender categories [41].

Despite no consistent relationship between household income and intention to place a bet on the Melbourne Cup (inferred from Statement 2), there was an association between full- or part-time employment and intention to place a bet on the Melbourne Cup. While Melbourne Cup day is a public holiday in Victoria, it is not in the rest of Australia, so this association may be due to either formal or informal office sweepstakes or other occupational social pressures to gamble.

Setting aside the Gamblers and the Paradoxical-voters, the remaining cluster showing the greatest intention of watching the Melbourne Cup and gambling on it are the Devotees, almost two thirds of whom agreed or strongly agreed they would watch the race and place a bet. Few of these Devotees report having either gambling or animal welfare concerns that interfere with their interest in the Cup, fewer even than the Gamblers cluster. It may be this group which is engaging with the Melbourne Cup as an iconic event, such that placing a bet is a part of fully participating in the ritual, and this might explain the unexpectedly even gender ratio (roughly 40% female to 60% male – or 51% female to 49% male if grouped with the Gamblers cluster) among this cluster.

Aligning with reports of high gambling rates among younger people than older people [42]), we found fewer people over 65 years in our Gamblers cluster than expected under a condition of no association between age and group, but more people over 65 years than expected amongst Devotees, fewer than expected among the Flaneurs but more among the Casuals. Indeed, the over 65 years group was one of two age groups with somewhat different from expected cluster distributions, with the other group being the 25-29 years group in which Gamblers were overrepresented and Casuals were somewhat underrepresented.

There are some indications in this study that interest in the Melbourne Cup is stronger for older age brackets than younger ones. Younger people were more likely to indicate that they had never been interested in the Melbourne Cup, and the Disapprover and the Flaneur clusters were both significantly younger than Devotees. The Paradoxical-voting cluster tended to be younger rather than older people and were more likely to be male.

Finally, with specific regard to gambling behavior, the poll did not differentiate between different forms of gambling. Research suggests that the new mode of internet gamblers differ in many ways from existing pre-gamblers [43]. They may also have different perceptions of animal cruelty and the welfare of Thoroughbred racehorses than offline gamblers.

5. Conclusions

Australia's most iconic horse race is also one of the most contentious events in Australia's public arena. The aims of this study were to discern relationships between the stated attitudes and behaviours of survey respondents and their demographic attributes, and to explore how attitudes toward the Melbourne Cup intersect with concerns about animal welfare and gambling.

Some associations were found between stated behaviours and demographics in relation to gender, employment status and age. Men were more likely to regularly bet on horse races, people with full or part-time employment were more likely to intend to place a bet on the Melbourne Cup and women were more likely to report lessening interest in the Melbourne Cup due to concerns for animal cruelty. Intentions to place a bet appeared to be unaffected be gender or income.

Six clusters were identified. Devotees (31%) were unlikely to identify as gamblers but were very interested in watching and betting on the Cup, showing consistency over time. Flaneurs (22%) were neither interested in betting in general, nor the Melbourne Cup in particular. Disapprovers (16%) were not regular gamblers and were unlikely to watch and/or place a bet on the Cup. They reflected dissenters who had never approved of the Melbourne Cup race as well as apostates who had lost interest and reported changing their behaviours over time. Casuals (14%) never bet on horse races but were very interested in watching the Melbourne Cup horse race. Gamblers (12%) were those for whom the Melbourne Cup was probably just another horse race they regularly bet on. Lastly, Paradoxical-voters (5%) were those who completed the survey but selected the first response available to them.

Devotees and Gamblers are the most enthusiastic gamblers on the Melbourne Cup, but at only 43%, they are outweighed by the disinterested Flaneurs, Disapprovers and Casuals who are unlikely to place a bet (52%). Still, the novelty of the Melbourne Cup seemed to inspire 31% of those who would not identify as gamblers to place a bet. If the future of Australia's Melbourne Cup horse race is dependent on the support of punters, findings suggest that whilst support seems solid, it may also be noncommittal and vulnerable to change. Indeed, this vulnerability could account for the 2019 Melbourne Cup experiencing a 24year record low in attendance following the airing of a damning television documentary about the industry's inability to track levels of 'wastage' or ensure animal welfare standards in abattoirs and slaughter houses [24]. As this study is based on data collected prior to the documentary, findings provide a foundation for future comparative research into the strength of punter commitment, vulnerability to negative press and the implications for the social license to race and gamble on horses.

Author Contributions: Conceptualization, P.M.; methodology, B.J.W.; formal analysis, B.J.W.; investigation, B.J.W.; resources, P.M.; data curation, B.J.W.; writing—original draft preparation, B.J.W.; writing—review and editing, B.J.W.; K.T. and P.M.; project administration, B.J.W.; funding acquisition, P.M.

Funding: The statistical analysis and the costs of publishing in open access were funded by the Sydney School of Veterinary Science and RSPCA Australia.

Acknowledgments: This work was a collaboration between the University of Sydney and RSPCA Australia. The authors thank Essential Media for making the data we have analysed available. The authors also thank Dr Bidda Jones for her advice and support.

Conflicts of Interest: The authors declare no conflict of interest.

References

1. Lagos E, Wrathall J, Alebaki M (2014) Motivations and expectations associated with attending major sporting events: The case of Melbourne cup. In: Chien PM, 2014: 963-968., editor. CAUTHE 2014: Tourism and Hospitality in the Contemporary World: Trends, Changes and Complexity. Brisbane: School of Tourism: The University of Queensland. pp. 963-968.

2. Cusack CM, Digance J (2009) The Melbourne Cup: Australian identity and secular pilgrimage. Sport in Society 12: 876-889.
3. Worthington A (2007) National exuberance: A note on the Melbourne cup effect in Australian stock returns. Economic Papers - Economic Society of Australia 26: 170.
4. Narayan PK, Smyth R (2004) The Race that Stops a Nation: The Demand for the Melbourne Cup. Economic Record 80: 193-207.
5. Winter C, Young W (2014) Fashion, fantasy and fallen horses: alternate images of thoroughbred racing. Annals of Leisure Research 17: 359-376.
6. McManus P, Albrecht G, Graham R (2013) The Global Horseracing Industry: Social, Economic, Environmental and Ethical Perspectives. New York: Routledge. 1-244 p.
7. Hitchens PL, Morrice-West AV, Stevenson MA, Whitton RC (2019) Meta-analysis of risk factors for racehorse catastrophic musculoskeletal injury in flat racing. Vet J 245: 29-40.
8. Crispe EJ, Secombe CJ, Perera DI, Manderson AA, Turlach BA (2019) Exercise-induced pulmonary haemorrhage in Thoroughbred racehorses: a longitudinal study. 51: 45-51.
9. Bell RJ, Kingston JK, Mogg TD, Perkins NR (2007) The prevalence of gastric ulceration in racehorses in New Zealand. N Z Vet J 55: 13-18.
10. Hood J, McDonald C, Wilson B, McManus P, McGreevy P (2017) Whip Rule Breaches in a Major Australian Racing Jurisdiction: Welfare and Regulatory Implications. Animals (Basel) 7.
11. Thompson K, McManus P, Stansall D, Wilson BJ, McGreevy PD (2020) Is Whip Use Important to Thoroughbred Racing Integrity? What Stewards' Reports Reveal about Fairness to Punters, Jockeys and Horses. Animals 10: 1985.
12. Tong L, Stewart M, Johnson I, Appleyard R, Wilson B, et al. (2020) A Comparative Neuro-Histological Assessment of Gluteal Skin Thickness and Cutaneous Nociceptor Distribution in Horses and Humans. Animals 10: 2094.
13. McGreevy PD, Franklin S (2018) Over 20% of Australian horses race with their tongues tied to their lower jaw. The Conversation.
14. Gainsbury SM, Russell AM, Hing N, Blaszczynski A (2018) Consumer engagement with and perceptions of offshore online gambling sites. New Media & Society 20: 2990-3010.
15. Duncan E, Graham R, McManus P (2018) 'No one has even seen… smelt… or sensed a social licence': Animal geographies and social licence to operate. Geoforum 96: 318-327.
16. Heleski C, Stowe CJ, Fiedler J, Peterson ML, Brady C, et al. (2020) Thoroughbred Racehorse Welfare through the Lens of 'Social License to Operate—With an Emphasis on a U.S. Perspective. Sustainability 12: 1706.
17. Graham R, McManus P (2016) Changing Human-Animal Relationships in Sport: An Analysis of the UK and Australian Horse Racing Whips Debates. Animals 6: 32.
18. Evans D, McGreevy P (2011) An investigation of racing performance and whip use by jockeys in Thoroughbred races. PloS one 6: e15622.
19. McGreevy PD, Hawson LA, Salvin H, McLean AN (2013) A note on the force of whip impacts delivered by jockeys using forehand and backhand strikes. Journal of Veterinary Behavior: Clinical Applications and Research 8: 395-399.
20. McGreevy PD, Griffiths MD, Ascione FR, Wilson B (2018) Flogging tired horses: Who wants whipping and who would walk away if whipping horses were withheld? PloS one 13: e0192843.
21. Hitchens PL, Blizzard CL, Jones G, Day LM, Fell J (2009) The incidence of race-day jockey falls in Australia, 2002-2006. MJA 190: 83-86.
22. Montoya D, McManus P, Albrecht G (2012) Jumping to Conclusions? Media Coverage of Jumps Racing Debates in Australia. Society and Animals 20: 273-293.
23. McManus P, Montoya D (2012) Toward new understandings of human–animal relationships in sport: a study of Australian jumps racing. Social & Cultural Geography 13: 399-420.
24. Meldrum-Hanna C (2019) The Final Race: The dark side of the horse racing industry. 7.30 Report.

25. McLean AN, McGreevy PD (2010) Ethical equitation: Capping the price horses pay for human glory. Journal of Veterinary Behavior 5: 203-209.
26. Jones B, McGreevy PD (2010) Ethical equitation: applying a cost-benefit approach. Journal of Veterinary Behavior: Clinical Applications and Research 5: 196-202.
27. McGreevy PD, McLean AN (2009) Punishment in horse-training and the concept of ethical equitation. Journal of Veterinary Behavior: Clinical Applications and Research 4: 193-197.
28. Cusack CM, Digance J (2013) The Melbourne Cup: Australian identity and secular pilgrimage. Australian Sport: Routledge. pp. 50-63.
29. Murphy K (2018) Labor pulls further in front of Coalition as Morrison's disapproval rating rises. The Guardian. Australia.
30. Venables WN, Ripley BD (2002) Modern Applied Statistics with S-PLUS. New York: Springer.
31. R Core Team (2016) R: A language and environment for statistical computing. Venna, Austria: R Foundation for Statistical Computing.
32. Maechler M, Rousseeuw P, Struyf A, Hubert M, Hornik K (2019) Cluster Analysis Basics and Extensions. R package version 2.1.0.
33. Markwell K, Firth T, Hing N (2017) Blood on the race track: an analysis of ethical concerns regarding animal-based gambling. Annals of Leisure Research 20: 594-609.
34. McManus P, Graham R (2014) Horse racing and gambling: comparing attitudes and preferences of racetrack patrons and residents of Sydney, Australia. Leisure Studies 33: 400-417.
35. Winter C (2017) Loving thoroughbreds to death: conflicting values in leisure experience. Annals of Leisure Research 20: 578-593.
36. Festinger L (1957) A theory of cognitive dissonance. Evanston, Illinois: Row, Peterson.
37. Arluke A, Sanders CR (1996) Regarding Animals. Philadelphia: Temple University Press.
38. Silberman MS (1988) Animal welfare, animal rights: the past, the present, and the 21st century. The Journal of Zoo Animal Medicine: 161-167.
39. Delfabbro P, King D (2012) Gambling in Australia: Experiences, problems, research and policy. Addiction 107: 1556-1561.
40. Munro L (2001) Caring about blood, flesh, and pain: Women's standing in the animal protection movement. Society & Animals 9: 43-61.
41. Herzog HA (2007) Gender Differences in Human–Animal Interactions: A Review. Anthrozoös 20: 7-21.
42. Hing N, Russell A, Tolchard B, Nower L (2016) Risk Factors for Gambling Problems: An Analysis by Gender. J Gambl Stud 32: 511-534.
43. Gainsbury S, Wood R, Russell A, Hing N, Blaszczynski A (2012) A digital revolution: Comparison of demographic profiles, attitudes and gambling behavior of Internet and non-Internet gamblers. Computers in Human Behavior 28: 1388-1398.

Supporting information

S1 Table. Demographic data from n= 1028 Survey Respondents indicating their agreement with six attitudes regarding the Annual Melbourne Cup Thoroughbred Horse Race. n= number of respondents selecting this demographic option, % percentage of respondents selecting this option. *Italicised responses* indicate respondent declined to answer and are not included in percentage calculations**.**

Figure 1

Click here to access/download;Figure;Figure1.tif

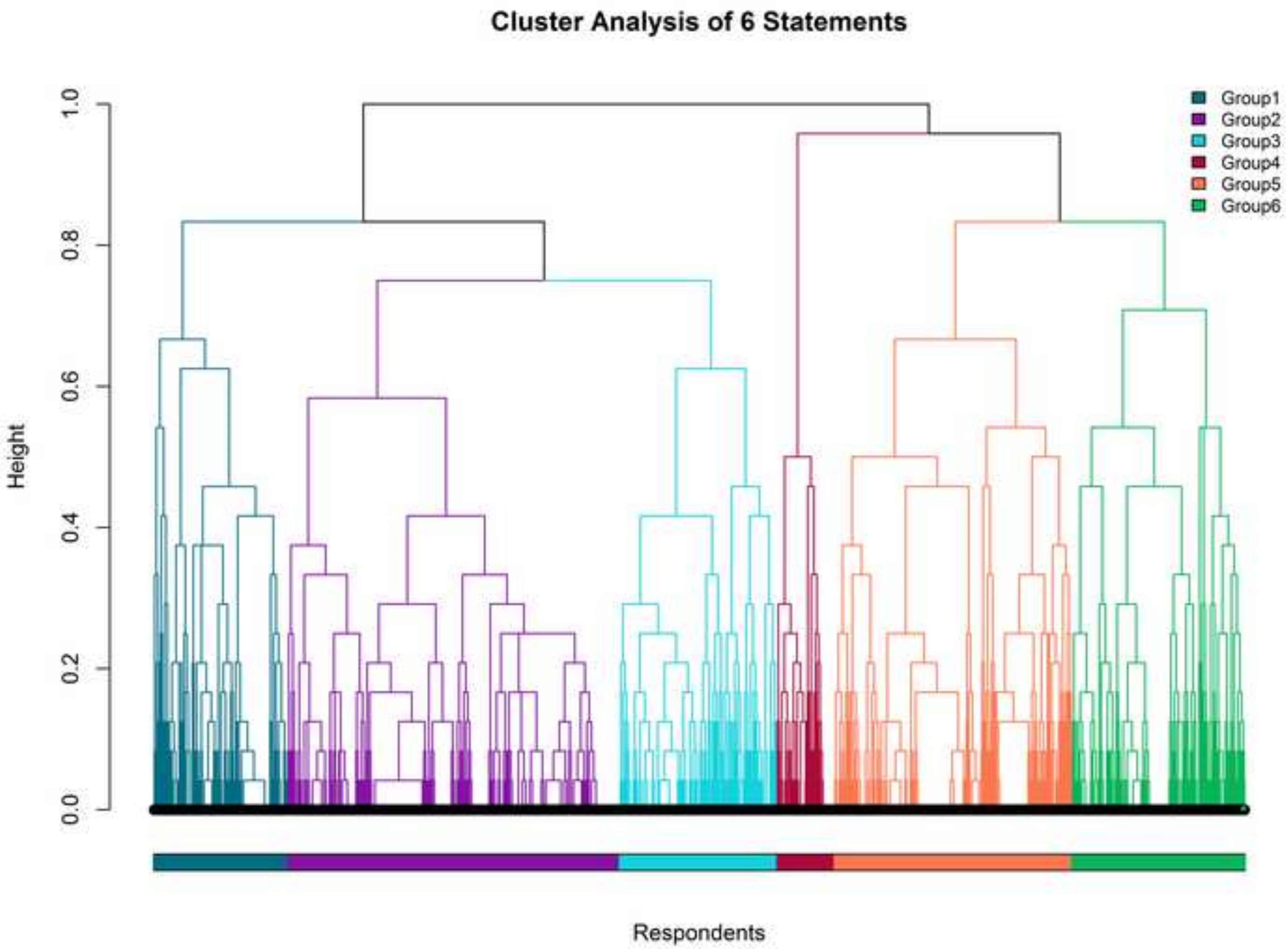

Figure 2

Click here to access/download;Figure;Figure2.tif

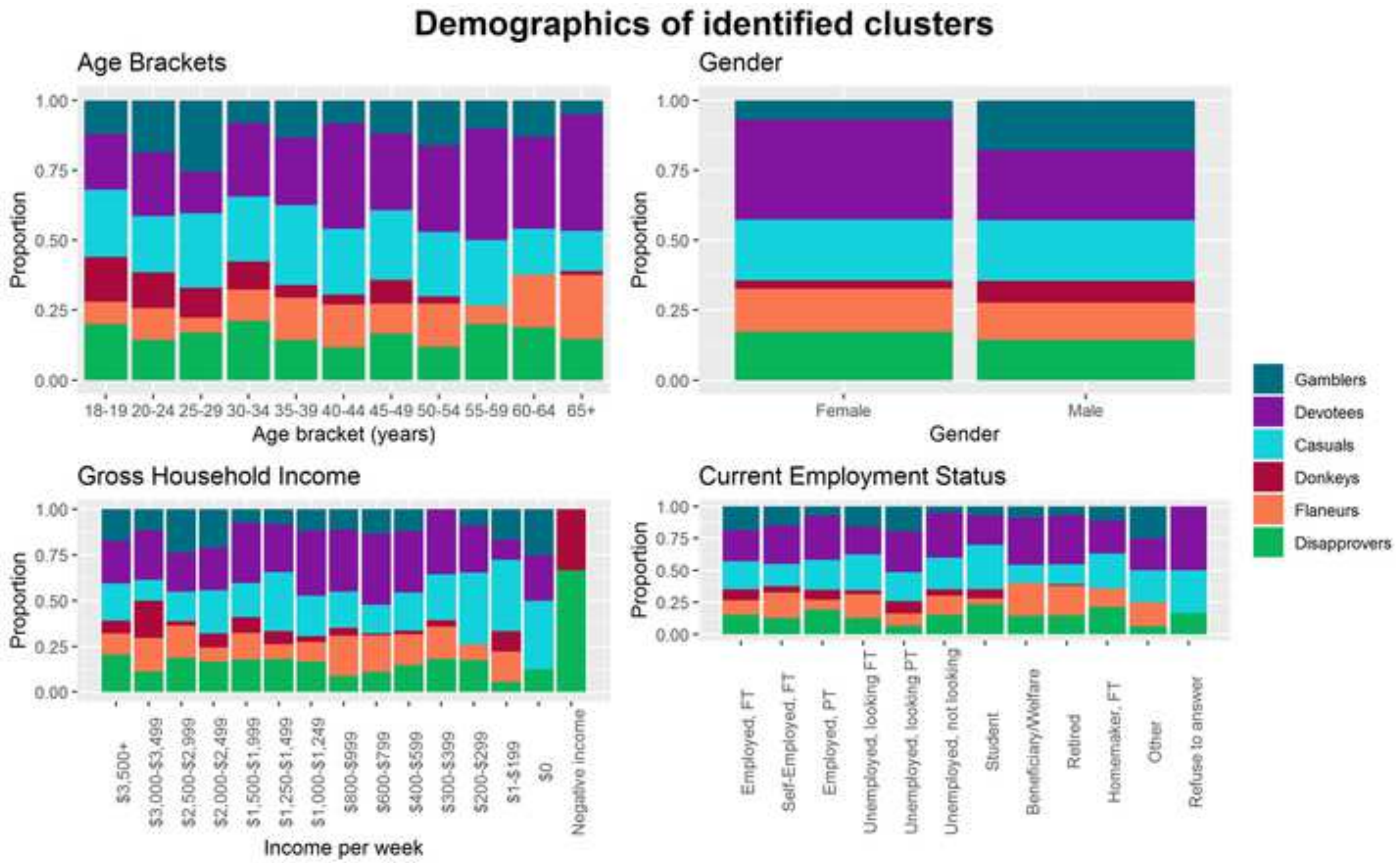

Click here to access/download
Supporting Information
S1_File.docx

The race that segments a nation: Findings from a convenience poll of attitudes toward the Melbourne Cup Thoroughbred horse race, gambling and animal cruelty

Formatted: Font: Not Bold

Formatted: Line spacing: Double

Bethany J. Wilson[1¶*], Kirrilly R. Thompson[2¶] and Paul D. McGreevy [1¶]

[1] Sydney School of Veterinary Science, Faculty of Science, University of Sydney, Sydney, NSW 2006, Australia;

[2] University of South Australia Business School 61 North Terrace, Adelaide SA 5000

* Correspondence: bethany.wilson@sydney.edu.au; Tel.: +61-7-3907-0998

¶ These authors contributed equally to this work

Formatted: Line spacing: Double

Formatted: Normal, Left

Received: date; Accepted: date; Published: date

Formatted: Line spacing: Double

Simple Summary: The data collection was performed by Essential Research, a division of Essential media, who collected data on respondents' gender, age, place of residence, weekly income, employment status and highest level of education, and asked for their level of agreement with the following six statements: *I regularly bet on horse races; I rarely bet on horse races but will be watching the Melbourne Cup and placing a bet; I will watch the Melbourne Cup but will not place a bet; I have never been interested in the Melbourne Cup; I have become less interested in the Melbourne Cup over recent years because of my concerns with gambling;* and *I have become less interested in the Melbourne Cup because of my concerns about animal cruelty*. The respondents' level of agreement with these statements revealed some

~~significant associations. For example, male respondents were at greater odds than average of agreeing with the statement: *I regularly bet on horse races*. Agreement with the statement: *I will watch the Melbourne Cup but will not place a bet* was more likely among the full time employed and those aged 20-24 years. Agreement with *I have never been interested in the Melbourne Cup* were highest among the relatively young age brackets of 25-34 years and lowest in the 60-64 years bracket. The strongest predictor of agreement with the statement: *I have become less interested in the Melbourne Cup over recent years because of my concerns with gambling* was level of education. Agreement with the statement: *I have become less interested in the Melbourne Cup because of my concerns about animal cruelty* was higher amongst female respondents and those in younger age brackets. These results show that Australian's attitudes to the Melbourne Cup, gambling and animal welfare relate to age, gender and level of education.~~

Abstract~~:~~

Formatted: Font: 18 pt

The annual Melbourne Cup Thoroughbred horse race has iconic status among many Australians but sits in the context of increasing criticism of the welfare of Thoroughbred racing horses and the ethics of gambling. Despite heated debates and protests playing out in the public domain, there is scant empirical research to document Australian attitudes to the Melbourne Cup, or horse racing more generally . Specifically, little is known about how support for or against the Melbourne Cup correlate with age, gender, income and level of education. To provide a more nuanced understanding of attitudes towards the cup beyond the rudimentary binaries of those who are 'for' or 'against' gambling and horse racing, the purpose of the study was to identify clusters of people with particular views. An opportunistic survey collected data on respondents' gender, age, place of residence, weekly income, employment status and highest level of education, and sought their level of agreement with six statements about the Melbourne Cup, gambling and animal cruelty. Ordinal logistic regression and Chi-square analysis were used to evaluate the age and gender of respondents in clusters

respectively. Agreement with the statements revealed some significant associations. Male respondents were at greater odds for agreement with the statement: *I regularly bet on horse races* (OR= 2.39; 95% CI=1.78-3.22) as were respondents aged 18-19 years (OR= 2.88; 95% CI=1.13-7.35) and 20-24 years (OR= 1.90; 95% CI 1.00-3.62) compared with the median 35-40 years age bracket. Agreement with the statement: I will watch the Melbourne Cup but will not place a bet was more likely among the full-time employed (OR= 1.60; 95% CI=1.10- 2.32), for those aged 20-24 years (OR=1.85; 95% CI=1.16-2.95). The odds of increasing agreement with the statement: *I have never been interested in the Melbourne Cup* were multiplied by 0.87 (95% CI=0.82-0.92) with each successive five-year age bracket. The most useful of the predictor variables for agreement was level of education. The odds of increasing with the statement: *I have become less interested in the Melbourne Cup over recent years because of my concerns with gambling* were multiplied by 1.09 (95% CI=1.02-1.15) for each increased level of education. Agreement with the statement: *I have become less interested in the Melbourne Cup because of my concerns about animal cruelty* was weaker amongst male respondents (OR= 0.62; 95% CI=0.48-0.80), and those in increasing age brackets (OR= 0.88; 95% CI=0.83-0.93). A series of six clusters were identified that show how certain attributes of respondents characterise their responses. The authors labelled these clusters “Devotees” (n=313; 30.4% of respondents), “Flaneurs” (n=244; 21.8% of respondents), “Disapprovers” (n=163; 15.9% of respondents), “Casuals” (n=148; 14.4% of respondents), “Gamblers” (n=126; 12.3% of respondents) and “Paradoxical-voters” (n=54; 5.3% of respondents). The implications for support of the Melbourne Cup are explored.

Keywords: horse-racing; gambling; animal welfare;

Formatted: Line spacing: Double

---

1. Introduction

Formatted: Font: 18 pt

The Melbourne Cup is a Thoroughbred horse race which takes place on the first Tuesday of November every year in the Melbourne suburb of Flemington, as the premier event of the Melbourne Spring Carnival [1]. First run in 1861, the race has become both a prominent part of the Australian national culture, listed with barbeques, football and ANZAC day as a core cultural symbol of Australian identity [2] and also a significant event on the global racing calendar, comparable to the Grand National, Kentucky Derby and Japan Cup [3,4].

Formatted: Line spacing: Double

Growing from the estimated crowd of four thousand who attended the first Melbourne Cup day [2] to turn-outs well in excess of 100,000 in the modern era [1,4], the event contributes an estimated AUD350 million to the state economy [4]. Annual betting of more than AUD105 million on this single race has been recorded, and global television audiences have been estimated at more than 1 billion [4]. In addition to gambling and the sport of horse-racing itself, since the 1960s the Melbourne Cup has also become intimately associated with fashion, and celebrity culture, creating another face of horse-racing with which the public can engage [2,5].

But despite its economic and social benefits, Thoroughbred racing in general[6], and the Melbourne Cup day in particular, potentially carry significant welfare costs to both horses and people. In addition to high profile deaths, such as the euthanasia of racehorse Cliffsofmoher following an injury early in the 2018 Melbourne Cup and the sudden death of Admire Rakti shortly after racing in 2014, In addition to the high profile deaths of Melbourne Cup runners on track or shortly after (7 horses since 2013), Thoroughbred racing is associated with widespread wastage~~Thoroughbred racing is associated with widespread wastage~~ [5] and acute and chronic pain from musculoskeletal injuries [7], pulmonary haemorrhages [8], gastric ulcers [9] and increasing public distaste for the use and consequence of equipment such as~~and as a consequence of the use of equipment such as~~whips [10-12] and tongue-ties [13]. Additionally, problem gambling is a widespread financial and mental health issue among Australians [14]. These concerns can have significant implications for the Thoroughbred

racing industry’s social license to operate [15,16] . Horse racing has been increasingly controversial in Australia over the past decades, mostly in relation to whip use [17-20] and injury and fatality rates in jumps racing [21-23]. More recently, there was a shared outcry from racing proponents and opponents alike in response to a *7.30 Report* exposé into the end of life for horse ‘wastage’ from the Australian Thoroughbred racing industry, particularly horses which had raced in New South Wales [24].

Formatted: Line spacing: Double

The ethical use of horses demands that we consider the welfare impact that horse-racing has on horses despite the economic and social benefits of horse-racing [25-27]. The Melbourne Cup, despite (or perhaps because of) its status as a cultural icon [28], is no exception.

Ahead of the 2018 Melbourne Cup, a commercial poll of adult Australians revealed that, when asked about horse-racing in general, 8% professed high interest in the sport, 20% reported moderate interest, while 70% said they had low or no interest [29]. However, although only 19% of the sample reported they regularly bet on horse-races, 38% said they would be watching the Melbourne Cup that year and would place a bet. Furthermore, 33% said they would be watching the event but not placing a bet. These data seem to confirm the iconic status that the Melbourne Cup has for many Australians [29].

Following the example offered by an earlier report on the use of polling data from a third party [20], the current study returns to the original data to explore relationships among these attitudes and respondents’ income, employment status, age and sex. It also explores how attitudes toward the Melbourne Cup intersect with concerns about animal welfare concerns and problem gambling.

2. Materials and mMethods

Formatted: Font: 18 pt

Formatted: Font: 18 pt

2.1 Data:

Formatted: Font: 16 pt, Bold, Not Italic

The data collection was performed by Essential Research, a division of Essential media, who, in addition to demographic data about gender, age, place of residence, weekly income, employment status and highest level of education, electronically polled respondents for their level of agreement with the following six statements:

1. I regularly bet on horse races
2. I rarely bet on horse races but will be watching the Melbourne Cup and placing a bet
3. I will watch the Melbourne Cup but will not place a bet
4. I have never been interested in the Melbourne Cup
5. I have become less interested in the Melbourne Cup over recent years because of my concerns with gambling
6. I have become less interested in the Melbourne Cup because of my concerns about animal cruelty

Formatted: Line spacing: Double

The questions were asked online as part of a larger omnibus of questions on a variety of topics allowing several days for survey completion under the supervisions of members of the Australian Market and Social Research Society (AMSRS), acting under a professional code of behavior. This was undertaken in the fortnight prior to 6th November 2018. We note that these data were collected well before the profile of Australian Racing was challenged by documentaries such as The Final Race (ABC TV's 7.30 Report, 17th of October 2019). While the process is intended to sample a random sample of the population, sampling errors due to lack of 100% response rate of invited respondents and gaps in coverage of the original pool from which invited respondents were sourced cannot be ruled out.

Formatted: Superscript

2.2.Analysis

Formatted: Font: 16 pt, Bold, Not Italic

Formatted: Font: 16 pt

2.2.1. Demographics

Formatted: Font: 14 pt, Bold

The association between respondents and respondent demographics were explored by ordinal logistic regression using the polr function of the MASS package in R [30,31].

The model used was

$Score_{ij}$ ~ $Gender_i$+ Age_i+ $Residence_i$+ $Income_i$+ $Employment_i$+ $Education_i$

Where $Score_{ij}$ = the Agreement Score (ie Strongly Disagree< Disagree<Don’t know<Agree< Strongly Agree) of Participant i to statement j (where j is one of statements 1-6 above); $Gender_I$ = Whether Participant i identified as “Male” or “Female”; Age_i = the age in years bracket nominated by Participant i for themselves; $Residence_i$ = Civic area where Participant i said that they lived; $Income_i$= Participant i’s nominated weekly income bracket; $Employment_i$ = Participant i’s nominated employment status; $Education_i$ = Participant I’s highest stated level of education.

2.2.2. Cluster a~~A~~nalysis

Formatted: Font: 14 pt, Bold

Formatted: Font: 14 pt, Bold

A hierarchical cluster analysis was performed on the Agreement Scores of the six statements (using Gower distance) with the daisy and hclust functions [32]. “Don’t know” was again placed centrally (i.e., Strongly Disagree< Disagree<Don’t know<Agree< Strongly Agree).

Ordinal logistic regression and Chi-square analysis were used to evaluate the age and gender of respondents in clusters respectively.

3. Results

3.1. Demographics

A total of 1028 respondents completed the survey, of whom 526 (51.2%) were female and 502 (48.8%) were male. Their agreement with the six statements about the Melbourne Cup, gambling and horse racing were stratified by gender (See Table 1).

Table 1. Ordinal agreement among 1028 respondents with six statements regarding the Melbourne Cup and horse-racing stratified by gender. ~~Frequency of response and (%) are offered.~~

**Statement**	**Strongly Disagree**	**Disagree**	**Don't Know**	**Agree**	**Strongly Agree**
S1: I regularly bet on horse races					
Female	348 (58%)	120 (56%)	16 (44%)	31 (28%)	11 (17%)
Male	255 (42%)	96 (44%)	20 (56%)	78 (72%)	53 (83%)
**Total**	**603 (59%)**	**216 (21%)**	**36 (4%)**	**109 (11%)**	**64 (6%)**
S2: I rarely bet on horse races but will be watching the Melbourne Cup and placing a bet					
Female	202 (54%)	98 (47%)	39 (54%)	119 (49%)	68 (51%)
Male	169 (46%)	111 (53%)	33 (46%)	123 (51%)	66 (49%)
**Total**	**371 (36%)**	**209 (20%)**	**72 (7%)**	**242 (24%)**	**134 (13%)**
S3: I will watch the Melbourne Cup but will not place a bet					
Female	189 (54%)	138 (52%)	47 (55%)	104 (45%)	48 (48%)
Male	158 (46%)	126 (48%)	39 (45%)	126 (55%)	53 (52%)
**Total**	**347 (34%)**	**264 (26%)**	**86 (8%)**	**230 (22%)**	**101 (10%)**

Formatted: Font: 18 pt

Formatted: Font: 16 pt, Bold, Not Italic

Formatted: Font: Bold

Formatted Table

Formatted: Font: Not Italic

Formatted: Font: Bold

Formatted: Font: Not Italic

Formatted: Font: Bold

Formatted: Font: Not Italic

Formatted: Font: Bold

S4: I have never been interested in the Melbourne Cup					
Female	154	182	25	78	87
Male	139	159	27	108	69
**Total**	**293 (29%)**	**341 (33%)**	**52 (5%)**	**186 (18%)**	**156 (15%)**
S5: I have become less interested in the Melbourne Cup over recent years because of my concerns with gambling					
Female	187	179	40	67	53
Male	164	154	38	102	44
**Total**	**351 (34%)**	**333 (32%)**	**78 (8%)**	**169 (16%)**	**97 (9%)**
S6: I have become less interested in the Melbourne Cup because of my concerns about animal cruelty					
Female	148	164	41	85	88
Male	178	168	35	78	43
**Total**	**326 (32%)**	**332 (32%)**	**76 (7%)**	**163 (16%)**	**131 (13%)**

Frequency of response and (%) are offered. Total rows sum to 100% horizontally and each sub category, divided by gender, will sum vertically".

Formatted: Font: Not Italic

Formatted: Font: Bold

Formatted: Font: Not Italic

Formatted: Font: Bold

Formatted: Font: Bold

Statement 1: "I regularly bet on horse races". Male respondents were at greater odds than the average respondent for agreement with this statement (OR= 2.39; 95% CI=1.78-3.22), as were respondents aged 18-19 years (OR= 2.88; 95% CI=1.13-7.35) and 20-24 years (OR= 1.90; 95% CI 1.00-3.62) compared with the median 35-40 years age bracket, and income earners in the bracket AUD$1-$199 per week (OR= 3.07; 95% CI 1.26-7.47).

In contrast, female respondents, respondents earning in the range of AUD$1,250-$1,499 per week (OR=0.54; 95% CI=0.32-0.91) and students (OR= 0.35; 95% CI=0.16-0.79) were at lesser odds than average for agreement. Respondents over 64 years of age (OR= 0.34 95% CI=0.16-0.73) were in less agreement than the median 35-40 years age bracket. Testing for an interaction between Gender and Age was found it to be not significant (p=0.43).

Statement 2: "I rarely bet on horse races but will be watching the Melbourne Cup and placing a bet". Unlike the first statement about habitual gambling, male respondents were not significantly more likely to say that, although they rarely bet on horse-racing, they would bet on the Melbourne Cup (LR χ^2= 0.0382, df=1 p=0.85).

Those aged 20-24 years showed higher odds of agreement (OR=1.73; 95% CI=1.10-2.71) with this statement than average whereas those aged 30-34 (OR=0.64; 95% CI=0.44-0.94) showed lower agreement. Respondents in the AUD$1,250-$1,499 per week income range were at lower odds of intending to gamble on the Melbourne Cup, as they also were at lower odds of regular gambling (OR= 0.58; 95% CI=0.38-0.89). Those employed full- (OR=1.98; 95% CI=1.36- 2.89) and part-time (OR=1.49; 95% CI=1.01-2.20) were at increased odds compared to the average of agreement for this statement.

Statement 3: "I will watch the Melbourne Cup but will not place a bet". Agreement with this statement was more likely among the full-time employed (OR= 1.60; 95% CI=1.10- 2.32), for those aged 20-24 years (OR=1.85; 95% CI=1.16-2.95) and less likely for those aged 50-54 years (OR= 0.67; 95% CI=0.47-0.96).

Statement 4: "I have never been interested in the Melbourne Cup". The odds of agreement with this statement were highest among the relatively young age brackets 25-29 years (OR= 2.04; 95% CI=1.40-2.97) and 30-34 (OR=1.61; 95% CI=1.10-2.35) and lowest in the older 60-64 years bracket (OR= 0.59; 95% CI=0.38- 0.92) compared to the average and in the 65 or older range 34 (OR=0.40; 95% CI=0.20-0.77) when compared to the 35-39 years range. This pattern is also true if age brackets are modelled ordinally with the odds of increasing agreement with the statement being multiplied by 0.87 (95% CI=0.82-0.92) with each successive five-year age bracket. Household income in the bracket

AUD$600-$799 per week also significantly reduced the odds of agreement compared to average (OR=0.59; 95% CI=0.38-0.92).

Statement 5: "I have become less interested in the Melbourne Cup over recent years because of my concerns with gambling". The most useful of the predictor variables for this statement was level of education. When education was modelled ordinally, the odds of increasing agreement with this ~~this~~ statement were multiplied by 1.09 (95% CI=1.02-1.15) for each increased level of education.

The odds of agreement were lowered for those in the income bracket of AUD$800-$999 per week. (OR= 0.55; 95% CI=0.36- 0.83).

Statement 6: "I have become less interested in the Melbourne Cup because of my concerns about animal cruelty". Male respondents (OR= 0.62; 95% CI=0.48-0.80), and increasing age brackets (OR= 0.88; 95% CI=0.83-0.93) were associated with lower odds of agreement with this statement as did the AUD$800-$999 per week household income bracket (OR=0.57; 95% CI=0.38-0.87).

3.2. Cluster analysis

Formatted: Font: 16 pt, Bold, Not Italic

Respondents were classified into six groups through agglomerative hierarchical clustering based on the Gower Distance. The hierarchical relationship between these six groups is shown by the dendrogram in Fig.~~ure~~ 1.

Fig~~ure~~ 1: Cluster analysis of respondents (n=1028) by ordinal agreement with six statements regarding 1028 respondents' attitudes to the Melbourne Cup.

Formatted: Font: Bold

The demographics of the clusters are shown in Fig.~~ure~~ 2.

Fig~~ure~~ 2: Demographics of respondents (n=1028) assigned to six clusters by ordinal agreement with six statements regarding attitudes to the Melbourne Cup.

Formatted: Font: Bold

3.2.1. Clusters

Formatted: Font: 14 pt, Bold

The six clusters are described below in order from most to least represented within the sample.

"Devotees". This cluster included 313 (30.4%) respondents. These respondents did not report regular gambling on horse-races (99.7% disagree or strongly disagree with "I regularly bet on horses"). Nevertheless, they showed very high interest in the Melbourne Cup (99.4% disagreed or strongly disagreed with "I have never been interested in the Melbourne Cup") and many planned to bet on it (63.6% agreed or strongly agreed with "I rarely bet on horse races but will be watching the Melbourne Cup and placing a bet"). Very few of this group reported reduced interest in the Cup due to gambling or welfare concerns (99.4% disagreed or strongly disagreed with "I have become less interested in the Melbourne Cup over recent years because of my concerns with gambling "; 97.8% disagreed or strongly disagreed with "I have become less interested in the Melbourne Cup over recent years because of my concerns about animal cruelty "). Women were over-represented among Devotees (χ^2 =7.2755, df = 1, p-value = 0.007).

"Flaneurs". This cluster included 224 (21.8%) respondents. Flaneurs did not report high rates of regular gambling on horse races (82.6% disagreed or strongly disagreed with "I regularly bet on horses") and they reported relatively low intention of watching the Melbourne Cup and placing a bet

(16.1% agreed or strongly agreed with "I rarely bet on horse races but will be watching the Melbourne Cup and placing a bet". They showed relatively low interest in the Melbourne Cup (79 % agreed or strongly agreed with "I have never been interested in the Melbourne Cup", and only 3.6% disagreed or strongly disagreed). Few agreed or strongly agreed to having reduced interest in the Melbourne Cup due to concerns about gambling (6.7%), but more reported reduced interest due to animal welfare concerns (17.9%). Neither women nor men were significantly over-represented but respondents in this cluster were younger than Devotees (-0.70, SE=0.15, p<0.01).

"Disapprovers'". This cluster included 163 (15.9%) respondents. Disapprovers did not report regular gambling on horse races (98.2% disagreed or strongly disagreed with "I regularly bet on horses"). Less than a quarter of this group agreed that they were planning to watch the Cup, with (22.1%) or without betting (16.0%). Neither women nor men were significantly over-represented but respondents in this cluster were younger than Devotees (-0.49, SE=0.17, p<0.01). Some Disapprovers revealed apostatic views. They reported the greatest loss of interest in the Melbourne Cup due to moral and ethical concerns; 89.0% reported lessened interest due to concerns with gambling, and 74.2% due to concerns with animal cruelty. A reasonable number of respondents in this cluster revealed dissenting views, as 35.6% disagreed or strongly disagreed that they have never been interested in the Melbourne Cup.

"Casuals". This cluster included 148 (14.4%) respondents. Like the Devotees, these respondents did not report regular gamblers on horse races (100% disagree or strongly disagree with "I regularly bet on horses"). Nonetheless, they did show high interest in the Melbourne Cup (89.2 % disagreed or strongly disagreed with "I have never been interested in the Melbourne Cup"), but they do not generally plan to bet on it (86.5% agreed or strongly agreed with "I will watch the Melbourne Cup but will not place a bet"). About a third of these respondents reported reduced interest in the Cup due to concerns about animal welfare (33.8%) and slightly fewer due to concerns about gambling (31.1%).

This cluster was not significantly older or younger than the Devotees and neither women nor men were overrepresented.

"Gamblers". This cluster included 126 (12.3%) respondents. Gamblers tended to report high levels of betting on horses in general (88.1% agree or strongly agree with "I regularly bet on horses"). They showed high interest in the Melbourne Cup (94.4% disagreed or strongly disagreed with "I have never been interested in the Melbourne Cup"). Few reported less interest in the Melbourne Cup due to concerns with gambling (7.14% agree or strongly agree). A little over a fifth reported (21.43%) less interest in the Melbourne Cup due to animal welfare concerns. Men were over-represented among Gamblers (χ^2=22.043, df=1, p-value <0.001) and were younger (-0.95, SE=0.19, p <0.01) than the Devotees.

"Paradoxical-voters" This cluster included 54 (5.3%) respondents. Paradoxical-voters provided contradictory responses throughout the survey, with a majority all agreeing or strongly agreeing with all six statements, despite the contradictions of doing so. Paradoxical-voters were overrepresented by males (χ^2 = 10.311, df = 1, p-value = 0.001) and were younger than Devotees (-1.78, SE=0.26, p <0.01)

4. Discussion

Formatted: Font: 18 pt

There are two main limitations to this study. First, the representativeness of the sample is limited by the convenience sampling strategy. However, it would not be unreasonable to assume that respondents had basic levels of English and online literacy as well as sufficient interest in the topics of gambling, racing and animal welfare to engage in the poll. Moreover, the polling company has a legitimate presence in Australia. Responses are made available weekly to online subscribers and a report is published in The Guardian Australia newspaper.

Second, the validity of the data is limited by some presumptuous wording of the survey statements. Whilst data were provided by a reputable independent research company, they were collected for a different aim than that discussed in this study. The six statements to which respondents indicated their agreement, disagreement and unsureness were designed to provide high rates of completion. For the purposes of this study, the validity of the statements may have been lowered by their inclusion of a frequency in the question form or a presumed relationship between two variables.

For example, Statement 1 ("I regularly bet on horse races") would most likely provide data with higher validity around betting frequency if it had collected numerical data around the number of occasions during which someone had bet over a stated period of time. Statement 2 ("I rarely bet on horse races but will be watching the Melbourne Cup and placing a bet") would most likely provide data with a more valid reflection of the prevalence of those whose betting on the Melbourne Cup is atypical of their general betting behavior if it simply asked about the intention to watch the Cup and place a bet, and was compared with data from Statement 1. Moreover, the inclusion of 'watch' and 'place a bet' may have yielded different data to a question asking only about 'watching' or 'only about betting'. The separation of Statement 2 into those two variables would then have provided more valid data sought from Statement 3 ("I will watch the Melbourne Cup but will not place a bet").

Statements 4, 5 and 6 were about interest in the Cup but were limited to statements about never being interested (Statement 4) or about becoming less interested due to a) concerns with gambling (Statement 5) and b) concerns with animal cruelty (Statement 6). Certainly, attitudes towards gambling and animal cruelty are mutually inclusive in animal-based gambling [33,34]. Nonetheless, validity was lowered by Statement 4 not providing an ordinal scale for level of interest and Statements 5 and 6 providing two pre-specified reasons for lowered interest.

The caveat in Statement 5 around gambling did not specify 'problem gambling', hence it is unclear what kind of gambling was most likely to be under consideration when responses were provided by respondents.

The caveat in Statement 6 around animal cruelty concerns may account for over or under-emphasis on gambling or animal cruelty depending on how a participant prioritized the reasons for their declining interest compared to their declining interest which may have been for other reasons (such as boredom, politics, concerns with alcohol, reduced income, etc.). In particular, not all those who are against animal cruelty perceive horse racing as cruel [35]. How such people in our sample responded to the social desirability bias of not wanting to appear to tolerate animal cruelty versus any strong convictions that racing is not cruel, or resolved the cognitive dissonance [36] of being interested in – or betting on a sport that others consider cruel remains to be determined. Cognitive dissonance may even be particularly salient in this context given that human society is fraught with contradictory relationships to animals [37] and views range across spectrum from (at least) welfare to rights [38]. Finally, response to Statement 6 may have been different had the less provocative term 'welfare' been used instead of 'cruelty'.

The limitations imposed on the responses that respondents were able to provide should be taken into consideration in the interpretation of the data presented here. Moreover, our findings and presentation of clusters are not exhaustive. There are other perspectives and clusters in the sample and general population which are beyond the scope of this paper. However, the aim of this study was not to discuss data in positivistic terms of representativeness and statistical significance. That would be disingenuous given the aforementioned limitations in sampling and design. Rather, the aim of this study was to conduct a preliminary exploration of associations between demographic variables and attitudes, as well as to initiate a non-binary understanding of attitudes towards the Melbourne Cup, gambling and animal cruelty.

This study suggests that attitudes towards the Melbourne Cup varied among the Australian population and are much more complex that simple binary views of being for or against Thoroughbred horse racing, gambling or animal cruelty. Therefore, despite being collected outside of academia, the data provide an opportunity to consider an important question that otherwise might be difficult to attract funding support, given corporate and nationalistic interests.

In particular, data also illustrate how stated behaviours and opinions vary demographically, especially in relation to gender, employment status and age. Contextualising findings within the literature is problematic, given that most of the research on gambling relates to specific populations, problematic or pathological gambling, online technologies and risk taking and sensation seeking behaviours, and is somewhat dated [39]. Intra-data comparisons do, however, yield some interesting findings.

Our results revealed that men showed more agreement with Statement 1 ("I regularly bet on horse races"), thus identifying themselves as regular gamblers on horse-races. In fact, 76% of those who agreed and strongly agreed with this statement were male. However, there was no association between gender and Statement 2, with 35.6% female respondents, and a similar 38.2% of male respondents agreeing or strongly agreeing, that despite not regularly gambling on horse-racing, they intended to watch the Melbourne Cup and place a bet. These findings suggest that betting behavior around Australia's most iconic horse race is atypical from racehorse gambling behavior throughout the year and that the novelty of betting on the Melbourne Cup is salient to men and women alike.

Some gendered differences were identified in relation to reported losses of interest in the Melbourne Cup due to concerns for animal cruelty (Statement 6), which was higher amongst female respondents. This is consistent with a general trend that women tend to show more concern for animal welfare than men [40], although across research on this subject there appears to be more variation within than between gender categories [41].

Despite no consistent relationship between household income and intention to place a bet on the Melbourne Cup (inferred from Statement 2), there was an association between full- or part-time employment and intention to place a bet on the Melbourne Cup. While Melbourne Cup day is a public holiday in Victoria, it is not in the rest of Australia, so this association may be due to either formal or informal office sweepstakes or other occupational social pressures to gamble.

Setting aside the Gamblers and the Paradoxical-voters, the remaining cluster showing the greatest intention of watching the Melbourne Cup and gambling on it are the Devotees, almost two

thirds of whom agreed or strongly agreed they would watch the race and place a bet. Few of these Devotees report having either gambling or animal welfare concerns that interfere with their interest in the Cup, fewer even than the Gamblers cluster. It may be this group which is engaging with the Melbourne Cup as an iconic event, such that placing a bet is a part of fully participating in the ritual, and this might explain the unexpectedly even gender ratio (roughly 40% female to 60% male – or 51% female to 49% male if grouped with the Gamblers cluster) among this cluster.

Aligning with reports of high gambling rates among younger people than older people [42]), we found fewer people over 65 years in our Gamblers cluster than expected under a condition of no association between age and group, but more people over 65 years than expected amongst Devotees, fewer than expected among the Flaneurs but more among the Casuals. Indeed, the over 65 years group was one of two age groups with somewhat different from expected cluster distributions, with the other group being the 25-29 years group in which Gamblers were overrepresented and Casuals were somewhat underrepresented.

There are some indications in this study that interest in the Melbourne Cup is stronger for older age brackets than younger ones. Younger people were more likely to indicate that they had never been interested in the Melbourne Cup, and the Disapprover and the Flaneur clusters were both significantly younger than Devotees. The Paradoxical-voting cluster tended to be younger rather than older people and were more likely to be male.

Finally, with specific regard to gambling behavior, the poll did not differentiate between different forms of gambling. Research suggests that the new mode of internet gamblers differ in many ways from existing pre-gamblers [43]. They may also have different perceptions of animal cruelty and the welfare of Thoroughbred racehorses than offline gamblers.

5. Conclusions

Formatted: Font: 18 pt

Australia’s most iconic horse race is also one of the most contentious events in Australia’s ~~the~~ public arena. The aims of this study were to discern relationships between the stated attitudes and behaviours of survey respondents and their demographic attributes, and to explore how attitudes toward the Melbourne Cup intersect with concerns about animal welfare and gambling.

Some associations were found between stated behaviours and demographics in relation to gender, employment status and age. Men were more likely to regularly bet on horse races, people with full or part-time employment were more likely to intend to place a bet on the Melbourne Cup and women were more likely to report lessening interest in the Melbourne Cup due to concerns for animal cruelty. Intentions to place a bet appeared to be unaffected be gender or income.

Six clusters were identified. Devotees (31%) were unlikely to identify as gamblers but were very interested in watching and betting on the Cup, showing consistency over time. Flaneurs (22%) were neither interested in betting in general, nor the Melbourne Cup in particular. Disapprovers (16%) were not regular gamblers and were unlikely to watch and/or place a bet on the Cup. They reflected dissenters who had never approved of the Melbourne Cup race as well as apostates who had lost interest and reported changing their behaviours over time. Casuals (14%) never bet on horse races but were very interested in watching the Melbourne Cup horse race. Gamblers (12%) were those for whom the Melbourne Cup was probably just another horse race they regularly bet on. Lastly, Paradoxical-voters (5%) were those who completed the survey but selected the first response available to them.

Devotees and Gamblers are the most enthusiastic gamblers on the Melbourne Cup, but at only 43%, they are outweighed by the disinterested Flaneurs, Disapprovers and Casuals who are unlikely to place a bet (52%). Still, the novelty of the Melbourne Cup seemed to inspire 31% of those who would not identify as gamblers to place a bet. If the future of Australia’s Melbourne Cup horse race is dependent on the support of punters, findings suggest that whilst support seems solid, it may also be noncommittal and vulnerable to change. Indeed, this vulnerability could account for the 2019 Melbourne Cup experiencing a 24year record low in attendance following the airing of a damning

television documentary about the industry's inability to track levels of 'wastage' or ensure animal welfare standards in abattoirs and slaughter houses [24]. As this study is based on data collected prior to the documentary, findings provide a foundation for future comparative research into the strength of punter commitment, vulnerability to negative press and the implications for the social license to race and gamble on horses.

Author Contributions: Conceptualization, P.M.; methodology, B.J.W.; formal analysis, B.J.W.; investigation, B.J.W.; resources, P.M.; data curation, B.J.W.; writing—original draft preparation, B.J.W.; writing—review and editing, B.J.W.; K.T. and P.M.; project administration, B.J.W.; funding acquisition, P.M.

Funding: The statistical analysis and the costs of publishing in open access were funded by the Sydney School of Veterinary Science and RSPCA Australia.

Acknowledgments: This work was a collaboration between the University of Sydney and RSPCA Australia. The authors thank Essential Media for making the data we have analysed available. The authors also thank Dr Bidda Jones for her advice and support.

Conflicts of Interest: The authors declare no conflict of interest.

References

1. Lagos E, Wrathall J, Alebaki M (2014) Motivations and expectations associated with attending major sporting events: The case of Melbourne cup. In: Chien PM, 2014: 963-968., editor. CAUTHE 2014: Tourism and Hospitality in the Contemporary World: Trends, Changes and Complexity. Brisbane: School of Tourism: The University of Queensland. pp. 963-968.
2. Cusack CM, Digance J (2009) The Melbourne Cup: Australian identity and secular pilgrimage. Sport in Society 12: 876-889.
3. Worthington A (2007) National exuberance: A note on the Melbourne cup effect in Australian stock returns. Economic Papers - Economic Society of Australia 26: 170.
4. Narayan PK, Smyth R (2004) The Race that Stops a Nation: The Demand for the Melbourne Cup. Economic Record 80: 193-207.
5. Winter C, Young W (2014) Fashion, fantasy and fallen horses: alternate images of thoroughbred racing. Annals of Leisure Research 17: 359-376.
6. McManus P, Albrecht G, Graham R (2013) The Global Horseracing Industry: Social, Economic, Environmental and Ethical Perspectives. New York: Routledge. 1-244 p.
7. Hitchens PL, Morrice-West AV, Stevenson MA, Whitton RC (2019) Meta-analysis of risk factors for racehorse catastrophic musculoskeletal injury in flat racing. Vet J 245: 29-40.
8. Crispe EJ, Secombe CJ, Perera DI, Manderson AA, Turlach BA (2019) Exercise-induced pulmonary haemorrhage in Thoroughbred racehorses: a longitudinal study. 51: 45-51.

9. Bell RJ, Kingston JK, Mogg TD, Perkins NR (2007) The prevalence of gastric ulceration in racehorses in New Zealand. N Z Vet J 55: 13-18.
10. Hood J, McDonald C, Wilson B, McManus P, McGreevy P (2017) Whip Rule Breaches in a Major Australian Racing Jurisdiction: Welfare and Regulatory Implications. Animals (Basel) 7.
11. Thompson K, McManus P, Stansall D, Wilson BJ, McGreevy PD (2020) Is Whip Use Important to Thoroughbred Racing Integrity? What Stewards' Reports Reveal about Fairness to Punters, Jockeys and Horses. Animals 10: 1985.
12. Tong L, Stewart M, Johnson I, Appleyard R, Wilson B, et al. (2020) A Comparative Neuro-Histological Assessment of Gluteal Skin Thickness and Cutaneous Nociceptor Distribution in Horses and Humans. Animals 10: 2094.
13. McGreevy PD, Franklin S (2018) Over 20% of Australian horses race with their tongues tied to their lower jaw. The Conversation.
14. Gainsbury SM, Russell AM, Hing N, Blaszczynski A (2018) Consumer engagement with and perceptions of offshore online gambling sites. New Media & Society 20: 2990-3010.
15. Duncan E, Graham R, McManus P (2018) 'No one has even seen… smelt… or sensed a social licence': Animal geographies and social licence to operate. Geoforum 96: 318-327.
16. Heleski C, Stowe CJ, Fiedler J, Peterson ML, Brady C, et al. (2020) Thoroughbred Racehorse Welfare through the Lens of 'Social License to Operate—With an Emphasis on a U.S. Perspective. Sustainability 12: 1706.
17. Graham R, McManus P (2016) Changing Human-Animal Relationships in Sport: An Analysis of the UK and Australian Horse Racing Whips Debates. Animals 6: 32.
18. Evans D, McGreevy P (2011) An investigation of racing performance and whip use by jockeys in Thoroughbred races. PloS one 6: e15622.
19. McGreevy PD, Hawson LA, Salvin H, McLean AN (2013) A note on the force of whip impacts delivered by jockeys using forehand and backhand strikes. Journal of Veterinary Behavior: Clinical Applications and Research 8: 395-399.
20. McGreevy PD, Griffiths MD, Ascione FR, Wilson B (2018) Flogging tired horses: Who wants whipping and who would walk away if whipping horses were withheld? PloS one 13: e0192843.
21. Hitchens PL, Blizzard CL, Jones G, Day LM, Fell J (2009) The incidence of race-day jockey falls in Australia, 2002-2006. MJA 190: 83-86.
22. Montoya D, McManus P, Albrecht G (2012) Jumping to Conclusions? Media Coverage of Jumps Racing Debates in Australia. Society and Animals 20: 273-293.
23. McManus P, Montoya D (2012) Toward new understandings of human–animal relationships in sport: a study of Australian jumps racing. Social & Cultural Geography 13: 399-420.
24. Meldrum-Hanna C (2019) The Final Race: The dark side of the horse racing industry. 7.30 Report.
25. McLean AN, McGreevy PD (2010) Ethical equitation: Capping the price horses pay for human glory. Journal of Veterinary Behavior 5: 203-209.
26. Jones B, McGreevy PD (2010) Ethical equitation: applying a cost-benefit approach. Journal of Veterinary Behavior: Clinical Applications and Research 5: 196-202.
27. McGreevy PD, McLean AN (2009) Punishment in horse-training and the concept of ethical equitation. Journal of Veterinary Behavior: Clinical Applications and Research 4: 193-197.
28. Cusack CM, Digance J (2013) The Melbourne Cup: Australian identity and secular pilgrimage. Australian Sport: Routledge. pp. 50-63.
29. Murphy K (2018) Labor pulls further in front of Coalition as Morrison's disapproval rating rises. The Guardian. Australia.
30. Venables WN, Ripley BD (2002) Modern Applied Statistics with S-PLUS. New York: Springer.
31. R Core Team (2016) R: A language and environment for statistical computing. Venna, Austria: R Foundation for Statistical Computing.
32. Maechler M, Rousseeuw P, Struyf A, Hubert M, Hornik K (2019) Cluster Analysis Basics and Extensions. R package version 2.1.0.

33. Markwell K, Firth T, Hing N (2017) Blood on the race track: an analysis of ethical concerns regarding animal-based gambling. Annals of Leisure Research 20: 594-609.
34. McManus P, Graham R (2014) Horse racing and gambling: comparing attitudes and preferences of racetrack patrons and residents of Sydney, Australia. Leisure Studies 33: 400-417.
35. Winter C (2017) Loving thoroughbreds to death: conflicting values in leisure experience. Annals of Leisure Research 20: 578-593.
36. Festinger L (1957) A theory of cognitive dissonance. Evanston, Illinois: Row, Peterson.
37. Arluke A, Sanders CR (1996) Regarding Animals. Philadelphia: Temple University Press.
38. Silberman MS (1988) Animal welfare, animal rights: the past, the present, and the 21st century. The Journal of Zoo Animal Medicine: 161-167.
39. Delfabbro P, King D (2012) Gambling in Australia: Experiences, problems, research and policy. Addiction 107: 1556-1561.
40. Munro L (2001) Caring about blood, flesh, and pain: Women's standing in the animal protection movement. Society & Animals 9: 43-61.
41. Herzog HA (2007) Gender Differences in Human–Animal Interactions: A Review. Anthrozoös 20: 7-21.
42. Hing N, Russell A, Tolchard B, Nower L (2016) Risk Factors for Gambling Problems: An Analysis by Gender. J Gambl Stud 32: 511-534.
43. Gainsbury S, Wood R, Russell A, Hing N, Blaszczynski A (2012) A digital revolution: Comparison of demographic profiles, attitudes and gambling behavior of Internet and non-Internet gamblers. Computers in Human Behavior 28: 1388-1398.

Supporting information

S1 Table. Demographic data from n= 1028 Survey Respondents indicating their agreement with six attitudes regarding the Annual Melbourne Cup Thoroughbred Horse Race. n= number of respondents selecting this demographic option, % percentage of respondents selecting this option. *Italicised responses* indicate respondent declined to answer and are not included in percentage calculations**.**

Response to Reviewers

The authors would like to very much thank the Reviewers and Academic Editors for their very helpful suggestions regarding this manuscript. Please see our detailed response and a record of the changes made to the manuscript below.

1. Please ensure that your manuscript meets PLOS ONE's style requirements, including those for file naming. The PLOS ONE style templates can be found at https://journals.plos.org/plosone/s/file?id=wjVg/PLOSOne_formatting_sample_main_body.pdf and https://journals.plos.org/plosone/s/file?id=ba62/PLOSOne_formatting_sample_title_authors_affiliations.pdf

Following this guideline

- The simple summary has been removed
- Major Sections have been headed with Size 18 Bold
- Italic Face has been removed from Minor section headings and changed to Bold Size 16
- Figure citations have been changed to “Fig. 1” and “Fig. 2”.
- Figure Titles have been changed to Bold Face\
- Level 3 Headings have been changed to Bold Face Size 14
- Headings changed to sentence case
- Double spacing added
- Article Title Unbolded
- Pilcrows added to author names

2. In your Methods section, please provide additional information about the participant recruitment method and the demographic details of your participants. Please ensure you have provided sufficient details to replicate the analyses such as: a) the recruitment date range (month and year), b) a description of any inclusion/exclusion criteria that were applied to participant recruitment, c) a table of relevant demographic details, d) a statement as to whether your sample can be considered representative of a larger population, e) a description of how participants were recruited, and f) descriptions of where participants were recruited and where the research took place.

a) This has been added

"The fortnight prior to 6th November 2018"

b) The recruitment process is in commercial confidence, however all Essential Research staff hold Australian Market and Social Research Society (AMSRS) membership and are bound by professional codes of behaviour.

c) This table has been prepared (Supplementary Table 1)

d) The following statement has been added.

"While the process is intended to sample a random sample of the population, sampling errors due to lack of 100% response rate of invited respondents and gaps in coverage of the original pool from which invited respondents were sourced cannot be ruled out."

e) f) While the exact process is a matter of commercial confidence, it is understood that approximately 7000-8000 participants from a larger (~100,000) Australia-wide panel are randomly invited to participate in each omnibus online interview, resulting in a variable response rate which, in this case, was 1028. As stated, the questions were presented online, and the residential details of respondents are now included in Supplementary Table 1.

3. We note that you have stated that you will provide repository information for your data at acceptance. Should your manuscript be accepted for publication, we will hold it until you provide the relevant accession numbers or DOIs necessary to access your data. If you wish to make changes to your Data Availability statement, please describe these changes in your cover letter and we will update your Data Availability statement to reflect the information you provide.

Contrary to our initial hopes, we have not obtained permission to release the data, although we encourage, in good faith, other researchers to apply to *Essential Media* for access to this data as we did.

We have updated our cover letter accordingly to reflect this information. We kindly request that our Data Availability statement be amended as such.

4. PLOS requires an ORCID iD for the corresponding author in Editorial Manager on papers submitted after December 6th, 2016. Please ensure that you have an ORCID iD and that it is validated in Editorial Manager. To do this, go to 'Update my Information' (in the upper left-hand corner of the main menu), and click on the Fetch/Validate link next to the ORCID field. This will take you to the ORCID site and allow you to create a new iD or authenticate a pre-existing iD in Editorial Manager. Please see the following video for instructions on linking an ORCID iD to your Editorial Manager account: https://www.youtube.com/watch?v=_xcclfuvtxQ

This step has been completed.

Reviewers' comments:

1) **Reviewer 2 expresses concern about the "strength" of statements "animal cruelty" (Simple summary) And "significant welfare costs for horses and people" Although they later state that these are addressed in the discussion.**

The removal of the simple summary in accordance with the journal requirements has addressed this first concern. The second statement has been moderated by the inclusion of the word "potentially"

> "But despite its economic and social benefits, Thoroughbred racing in general, and the Melbourne Cup day in particular, ***potentially*** carry significant welfare costs to both horses and people."

As the reviewer notes, further discussion is to be found later in the manuscript.

2) **Reviewer 2 would like the following paper to be referenced https://www.mdpi.com/2071-1050/12/5/1706/htm**
Heleski, C.; Stowe, C.J.; Fiedler, J.; Peterson, M.L.; Brady, C.; Wickens, C.; MacLeod, J.N. Thoroughbred Racehorse Welfare through the Lens of 'Social License to Operate—With an Emphasis on a U.S. Perspective. *Sustainability* 2020, *12*, 1706.

This reference has been added (new Reference 14)

Two additional new references (new Reference 12, and new Reference 13) have also been added.

3) **The reviewer would like us to clarify that this data was collected prior to a major public expose in the materials and methods**

The following text has been added to Materials and Methods.

> *"We note that these data were collected well before the profile of Australian Racing was challenged by documentaries such as The Final Race (ABC TV's 7.30 Report, 17th October 2019)."*

4) **Reviewer would like options 5 and 6 from the list to align (I believe geometrically!) with 1 through 4 (Page 9)**

This has been done.

5) **The Reviewer notes that there is a word missing from "The association between respondents and were explored by ordinal logistic regression"**

The words "respondent demographics" have been inserted as follows.

> *"The association between respondents and* ***respondent demographics*** *were explored by ordinal logistic regression using the polr function of the MASS package in R [28,29]."*

6) **The Reviewer would like a clarification for the reader on the way that the sums total in Table 1 on Page 10. They suggest adding language like "total rows sum to 100% horizontally" and "each sub category, divided by gender, will sum vertically".**

To address this point the reviewers' suggested text has been added to the table legend and the total rows have been bolded to distinguish them visually.

7) **The Reviewer is uncertain of the validity and the utility of the median column in Table 1 and suggests deleting it.**

This change has been made.

8) **The Reviewer notes an extra "this" in the statement 5 text on Page 11 "When education was modelled ordinally, the odds of increasing agreement with this this statement were multiplied by 1.09 (95% CI=1.02-1.15) for each increased level of education."**

The extra 'this' has been deleted. Thank you.

9) **The Reviewer would like "in the public arena" to be changed to "in Australia's public arena" Page 15. First sentence of Conclusion.**

This change has been made.
