# Supplementary material for: The race that segments a nation: Findings from a convenience poll of attitudes toward the Melbourne Cup Thoroughbred horse race, gambling and animal cruelty
Source: PLoS One. 2021 Mar 24;16(3):e0248945. doi: 10.1371/journal.pone.0248945 (PMC7990293; doi:10.1371/journal.pone.0248945)
Supplement: S1 Table — n = number of respondents selecting this demographic option, % percentage of respondents selecting this option. Italicised responses indicate respondent declined to answer and are not included in percentage calculations. (DOCX) [file pone.0248945.s002.docx]

**S1 Table. Demographic data from n= 1028 Survey Respondents indicating their agreement with six attitudes regarding the Annual Melbourne Cup Thoroughbred Horse Race**. n= number of respondents selecting this demographic option, % percentage of respondents selecting this option. *Italicised responses* indicate respondent declined to answer and are not included in percentage calculations**.**

| **Gender** | n | % | **Employment** | n | % |
| --- | --- | --- | --- | --- | --- |
| Male | 502 | 48.8% | Full time employment | 334 | 32.7% |
| Female | 526 | 51.2% | Full Time self-employment | 40 | 3.9% |
|  |  |  | Part Time employment | 179 | 17.5% |
| **Weekly Household Income** |  |  | Unemployed looking for full time work | 32 | 3.1% |
| $3,500+ | 59 | 6.3% | Unemployed looking for part time work | 31 | 3.0% |
| $3,000-$3,499 | 44 | 4.7% | Not employed and not looking for work | 20 | 2.0% |
| $2,500-$2,999 | 80 | 8.5% | Student | 43 | 4.2% |
| $2,000-$2,499 | 90 | 9.6% | Beneficiary/Welfare | 35 | 3.4% |
| $1,500-$1,999 | 124 | 13.2% | Retired | 211 | 20.6% |
| $1,250-$1,499 | 84 | 9.0% | Looking after the house full time | 81 | 7.9% |
| $1,000-$1,249 | 95 | 10.1% | Other | 16 | 1.6% |
| $800-$999 | 91 | 9.7% | *Refuse to answer* | *6* |  |
| $600-$799 | 90 | 9.6% |  |  |  |
| $400-$599 | 101 | 10.8% | **Education** |  |  |
| $300-$399 | 28 | 3.0% | Year 10 or below | 120 | 11.7% |
| $200-$299 | 23 | 2.5% | Year 11 or equivalent | 56 | 5.5% |
| $1-$199 | 18 | 1.9% | Year 12 or equivalent | 154 | 15.0% |
| No income | 8 | 0.9% | Still attending School | 4 | 0.4% |
| Negative income | 3 | 0.3% | Trade certificate or apprenticeship | 95 | 9.3% |
| *Prefer not to say* | *90* |  | Diploma/Certificate | 260 | 25.4% |
|  |  |  | Bachelors degree | 222 | 21.7% |
| **Residence** |  |  | Post Graduate Qualifications | 110 | 10.7% |
| Melbourne | 190 | 18.5% | Other | 3 | 0.3% |
| VIC (other) | 63 | 6.1% | *Prefer not to answer* | *4* |  |
| ACT | 15 | 1.5% |  |  |  |
| Adelaide | 60 | 5.8% | **Age** |  |  |
| SA (other) | 27 | 2.6% | 18-19 | 25 | 2.4% |
| Brisbane | 100 | 9.7% | 20-24 | 70 | 6.8% |
| QLDother | 100 | 9.7% | 25-29 | 94 | 9.1% |
| Darwin | 2 | 0.2% | 30-34 | 99 | 9.6% |
| NT (other) | 1 | 0.1% | 35-39 | 112 | 10.9% |
| Hobart | 11 | 1.1% | 40-44 | 85 | 8.3% |
| TAS (other) | 23 | 2.2% | 45-49 | 84 | 8.2% |
| Perth | 80 | 7.8% | 50-54 | 117 | 11.4% |
| WA (other) | 18 | 1.8% | 55-59 | 60 | 5.8% |
| Sydney | 212 | 20.6% | 60-64 | 85 | 8.3% |
| NSW (other) | 126 | 12.3% | 65+ | 197 | 19.2% |
